# Radiolabeling of Human Serum Albumin With Terbium-161 Using Mild Conditions and Evaluation of *in vivo* Stability

**DOI:** 10.3389/fmed.2021.675122

**Published:** 2021-08-18

**Authors:** Irwin Cassells, Stephen Ahenkorah, Andrew R. Burgoyne, Michiel Van de Voorde, Christophe M. Deroose, Thomas Cardinaels, Guy Bormans, Maarten Ooms, Frederik Cleeren

**Affiliations:** ^1^Radiopharmaceutical Research, Department of Pharmacy and Pharmacology, KU Leuven, Leuven, Belgium; ^2^Belgian Nuclear Research Centre (SCK CEN), Institute for Nuclear Materials Science, Mol, Belgium; ^3^Nuclear Medicine, University Hospitals Leuven, Nuclear Medicine & Molecular Imaging, Department of Imaging and Pathology, KU Leuven, Leuven, Belgium; ^4^Department of Chemistry, KU Leuven, Leuven, Belgium

**Keywords:** terbium-161, radiopharmaceutical, radiolabeling, TRNT, bio-conjugation

## Abstract

Targeted radionuclide therapy (TRNT) is a promising approach for cancer therapy. Terbium has four medically interesting isotopes (^149^Tb, ^152^Tb, ^155^Tb and ^161^Tb) which span the entire radiopharmaceutical space (TRNT, PET and SPECT imaging). Since the same element is used, accessing the various diagnostic or therapeutic properties without changing radiochemical procedures and pharmacokinetic properties is advantageous. The use of (heat-sensitive) biomolecules as vector molecule with high affinity and selectivity for a certain molecular target is promising. However, mild radiolabeling conditions are required to prevent thermal degradation of the biomolecule. Herein, we report the evaluation of potential bifunctional chelators for Tb-labeling of heat-sensitive biomolecules using human serum albumin (HSA) to assess the *in vivo* stability of the constructs. *p*-SCN-Bn-CHX-A”-DTPA, *p*-SCN-Bn-DOTA, *p*-NCS-Bz-DOTA-GA and *p*-SCN-3*p*-C-NETA were conjugated to HSA via a lysine coupling method. All HSA-constructs were labeled with [^161^Tb]TbCl_3_ at 40°C with radiochemical yields higher than 98%. The radiolabeled constructs were stable in human serum up to 24 h at 37°C. ^161^Tb-HSA-constructs were injected in mice to evaluate their *in vivo* stability. Increasing bone accumulation as a function of time was observed for [^161^Tb]TbCl_3_ and [^161^Tb]Tb-DTPA-CHX-A”-Bn-HSA, while negligible bone uptake was observed with the DOTA, DOTA-GA and NETA variants over a 7-day period. The results indicate that the *p*-SCN-Bn-DOTA, *p*-NCS-Bz-DOTA-GA and *p*-SCN-3*p*-C-NETA are suitable bifunctional ligands for Tb-based radiopharmaceuticals, allowing for high yield radiolabeling in mild conditions.

## Introduction

Terbium is an emerging theranostic element that has four medically relevant radioisotopes (^149^Tb, ^152^Tb, ^155^Tb and ^161^Tb) (1). For instance, ^152^Tb (β^+^ emitter, t_1/2_ = 17.5 h, E$\beta_{\text{average}}^{+}$ = 1.140 MeV) and ^155^Tb (EC, γ emitter, t_1/2_ = 5.32 days, Eγ = 0.105 MeV) can be used in diagnostic applications, such as positron emission tomography (PET) and single photon emission computed tomography (SPECT), respectively (1, 2). ^149^Tb (α emitter, t_1/2_ = 4.12 h, Eα = 3.97 MeV) and ^161^Tb (β^−^ emitter, t_1/2_ = 6.90 days, E$\beta_{\text{average}}^{-}$ = 0.154 MeV) on the other hand can be applied in targeted alpha (α) and beta (β) therapy, respectively (3–5). Once radiolabeling is optimized for a single radionuclide, the same protocol can be used to incorporate other terbium isotopes.

Because of its similar decay process and physical half-life, ^161^Tb is often considered a promising alternative for ^177^Lu (β^−^ emitter, t_1/2_ = 6.7 days, E$\beta_{\text{average}}^{-}$ = 0.134 MeV) (4, 6), which has become the golden standard in beta therapy since the clinical approval of Lutathera® ([^177^Lu]Lu-DOTATATE) for the treatment of neuroendocrine tumors (7, 8). In a study by Müller et al. (9), a direct comparison of the therapeutic effect of the two radioisotopes was investigated. It was concluded that [^161^Tb]Tb-cm09 had increased potency compared to [^177^Lu]Lu-cm09 (9). In a follow-up study using a prostate specific membrane antigen (PSMA) targeting radiopharmaceutical, increased survival of prostate cancer tumor-bearing mice was observed when treated with [^161^Tb]Tb-DOTA-PSMA-617 compared to [^177^Lu]Lu-DOTA-PSMA-617 (3). It has been postulated that the added efficacy of ^161^Tb is due to the additional therapeutic effect of Auger/conversion electrons (E_e−_ = 36 keV) (3, 4, 9, 10). In addition to the therapeutic power of ^161^Tb, there is co-emission of gamma radiation (Eγ = 49 keV, I = 17.0%; Eγ = 75 keV, I = 10.2%) (4), which can be used for SPECT imaging [similar to ^177^Lu (Eγ = 113 keV, I = 6.17%, Eγ = 208 keV, I = 10.36%)] (11, 12), as illustrated with the first-in-human application of [^161^Tb]Tb-DOTATOC (13).

Radiolabeling of a vector molecule with a terbium isotope is accomplished through specific bifunctional chelating agents (1). These bifunctional agents are often coupled in a non-site-specific manner to biological vector molecules through reactions with free lysines (e.g., using bifunctional chelators containing activated esters or isothiocyanate groups) or coupled site-specifically on cysteine groups (e.g., using thiol-maleimide chemistry) (14). Chelating agents, such as the macrocyclic 1,4,7,10-tetraazacyclododecane-1,4,7,10-tetraacetic acid (DOTA) and acyclic diethylene-triamine-pentaacetic acid (DTPA) type ligands exhibit high affinity for lanthanides and have been used extensively for decades by the radiopharmaceutical community (2, 15–17). Ideally, to allow radiolabeling of heat-sensitive vector molecules, ligands should combine the stable nature of the DOTA-metal bond and the fast reaction kinetics of DTPA chelators (18). In recent years, ligands such as [4-[2-(bis-carboxymethyl-amino)-ethyl]-7-carboxymethyl-[1, 4, 7] triazonan-1-yl]-acetic acid (NETA) have recently also received attention, as they have a hybrid structure in between the flexible DTPA framework and rigid DOTA core (19, 20).

**Figure 1:**
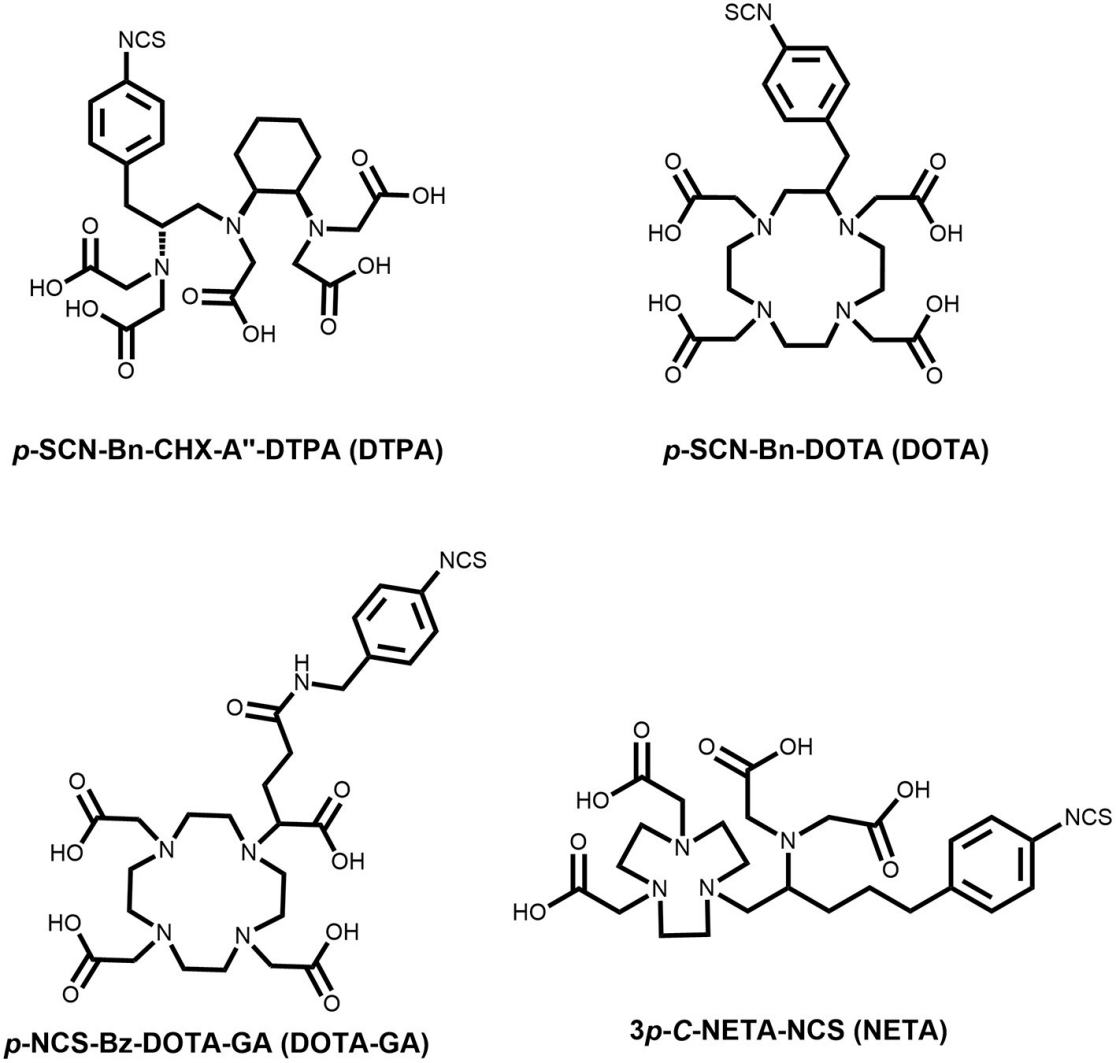
Chemical structure of bifunctional chelators used in this study.

Up until now, most terbium-labeling reactions were reported with ligands and peptides which are compatible with high radiolabeling temperatures (3, 9). Biomolecule-based radionuclide therapies, e.g., using trastuzumab for cancers with overexpression of human epidermal growth factor receptor-2 (HER-2), have paved the way for a more targeted approach to theranostics (21). Radiolabeling with terbium, and most of the other f-block elements, were performed at temperatures (>90°C), well-exceeding the temperatures compatible with heat-sensitive biomolecules such as monoclonal antibodies and antibody fragments (22). In this study, we developed a mild radiolabeling protocol (reaction temperature of 40°C in an aqueous buffer), with a series of commonly used bifunctional ligands (Figure 1). We then used human serum albumin (HSA, 66.5 kDa) as a model protein to assess the *in vitro* and *in vivo* stability of the corresponding ^161^Tb-labeled HSA conjugates. The high solubility, stability and plasma half-life of approximately 16-18 h make HSA the ideal vector to evaluate the stability of the ^161^Tb-chelates *in vivo*.

## Materials and Methods

### Materials

Reagents, unless specified otherwise, were purchased from Sigma-Aldrich (Bornem, Belgium) and used without further purification. Solvents were purchased from VWR (Leuven, Belgium) or Sigma-Aldrich, and used without further purification. *p*-SCN-Bn-CHX-A”-DTPA (**DTPA**) and *p*-SCN-Bn-DOTA (**DOTA**) were purchased from Macrocyclics, Inc. (Texas, USA), and *p*-NCS-Bz-DOTA-GA (**DOTA-GA**) was purchased from CheMatech (Dijon, France), and used without further purification. 3*p*-C-NETA-NCS (**NETA**) was synthesized and characterized according to literature methods (19). All radiolabeling buffers were stirred with chelex [Chelex 100 sodium form (50–100 mesh, Sigma Aldrich)], to remove trace metals, for 15 min and then filtered to remove the chelex beads. All solutions were degassed and filtered before use.

### Animals

Healthy albino Naval Medical Research Institute (NMRI) mice (age: 6–8 weeks, Envigo, Gannat, France) were housed in individually ventilated cages (IVC) in a regulated environment (22°C, humidity, 12 h day/night cycle), with food and water. Animal experiments were conducted according to the Belgian code of practice and use of animal experiments were approved by the ethical committee for animal care from KU Leuven.

### Instrumentation and Characterization

Mass spectra were recorded on an ultra-high-resolution time-of-flight mass spectrometer with electrospray ionization (ESI) (Bruker MaXis Impact, Bremen, Germany), coupled to a Dionex Ultimate 3,000 UPLC System (Thermo Fisher Scientific, USA). Quantification of protein concentration was determined using a microvolume UV-Vis spectrophotometer (NanoDrop One, Thermo Fisher Scientific). Quality assurance of the derivatized HSA constructs and ^161^Tb-HSA constructs were carried out with size-exclusion chromatography (SEC) using a Superdex 200 10/300 GL column (GE Healthcare Bio-Science AB, Uppsala, Sweden), eluted with a sodium phosphate buffer (0.15 M sodium chloride, 0.01 M phosphate, pH 7.4, Thermo Fisher) at a flow rate of 0.75 mL/min. The column effluent was passed through a UV detector (2998 PDA detector, Waters) in series with a 3-inch NaI(Tl) radioactivity detector. Gamma counting was performed on a Wizard^2^ 3470 [crystal: NaI (Tl), 50 mm in height, 32 mm in diameter, dead time 2.5 μs; Perkin Elmer, Germany], with a detection profile referenced for ^161^Tb decay (4). Counts were corrected for background radiation, physical decay and counter dead time.

### Production of [^161^Tb]TbCl_3_

[^161^Tb]TbCl_3_ was produced using a method adapted from literature (4). In brief, enriched ^160^Gd_2_O_3_ (1.0 mg, 98.2 %, Isoflex USA) was loaded as a nitrate salt into a quartz ampoule and sealed. The ampoule was sealed inside an aluminum capsule and was irradiated for 10 days in the BR2 Reactor at the Belgian Nuclear Research Centre (SCK CEN) at a thermal neutron flux of 3.0 x 10^14^ n/cm^2^/s. Following the irradiation and subsequent cooling for 5 days, the irradiated material was dissolved in trace-metal grade water. High-pressure ion chromatography (HPIC, Shimadzu), with a strong cation exchange column (ø: 6 mm, l: 50 mm, Shodex IC R-621), was used to separate the [^161^Tb] from the [^160^Gd] target matrix by elution with α-hydroxyisobutyric acid, with ammonium hydroxide (trace-metal grade) (added to adjust to pH 4.5). The collected fractions containing [^161^Tb] were combined and concentrated by loading them onto a column packed with extraction resin (ø: 2.1 mm, l: 30 mm, LN3, TrisKem International) and eluted with 50 mM hydrochloric acid (trace-metal free). The isolated solutions of [^161^Tb]TbCl_3_ had a radionuclidical purity of 99.998% (determined by gamma spectroscopy), a concentration of ~ 0.99 MBq/μL, and specific activity of ~ 3.6 TBq/mg.

### Human Serum Albumin (HSA) Ligand Constructs

A five-molar excess of bifunctional ligand (3 μmol) in 200 μl of a sodium bicarbonate solution (0.05 M, pH 8.5, 1.5 % DMSO) was added dropwise to a stirring solution of human serum albumin (400 μL, 0.6 μmol, CAF-DCF, Brussels, Belgium) in sodium bicarbonate (0.05 M, pH 8.5) in a LoBind vial (Eppendorf, Aarschot, Belgium). The mixture was then stirred for 2 h at room temperature and the conjugate was purified using a size exclusion chromatography cartridge (PD-10 column, GE Healthcare Bio-Science AB, Uppsala, Sweden) eluted with sodium acetate buffer (0.1 M, pH 4.7). The concentration of the HSA-ligand construct in the final reaction product was determined using spectrophotometry at 280 nm (NanoDrop® One, Thermo Fisher Scientific), with ε = 35,700 L/mol/cm and M = 66,477 g/mol. The purified product was analyzed using SEC using the method described above. UV detection of the eluate was performed at 280 nm. The number of chelators per protein was estimated by ESI-TOF-HRMS analysis considering the most abundant peak. The system was equipped with a Waters Acquity UPLC BEH C18 column (1.7 μm 2.1 × 50 mm, Waters, Milford, USA) using a gradient at a flowrate of 0.6 mL/min with mobile phase A: H_2_O, 0.1% HCOOH and mobile phase B: acetonitrile, 0.1 % HCOOH. The column was heated to 40°C. The elution gradient was: 0-2 min: 95% A; 2–8 min: from 95% A to 5% A; 8–10 min: 5% A; 10–12 min: from 5% A to 95% A. Calculated molecular ion mass values were obtained using Compass Isotope Pattern (version 3.2, Bruker) software. **HSA-DTPA**: ESI-MS *m/z* (decon.) calculated for HSA [66,477.96] + C_52_H_68_N_8_O_20_S_2_·H_6_Cl_6_ [1,296.33]: 67,852.22. Found: 67,852.20 (81.9 %). **HSA-DOTA**: ESI-MS *m/z* (decon.) calculated for HSA [66,477.96] + C_48_H_66_N_10_O_16_S_2_·H_3_Cl_3_ [1,210.34]: 67 688.30. Found: 67 687.74 (31.4 %). **HSA-DOTA-GA**: ESI-MS *m/z* (decon.) calculated for HSA [66,485.42] + C_54_H_76_N_12_O_18_S_2_·C_2_H_4_O_4_ [1,336.50]: 67,821.91. Found: 67,821.87 (95.2 %). **HSA-NETA**: ESI-MS *m/z* (decon.) calculated for HSA [66,457.96] + C_52_H_74_N_10_O_16_S_2_·C_2_O_5_H_6_ [1,268.49]: 67,726.46. Found: 67,726.63 (80.1 %).

### Radiolabeling Studies With [^161^Tb]TbCl_3_

Optimization of radiolabeling conditions: [^161^Tb]TbCl_3_ (0.2 MBq, 10 μL, 50 mM HCl) was added to 90 μL of a solution with different quantities of the ligand (**DTPA**, **DOTA**, **DOTA-GA** or **NETA**, 0.1-1.0 nmol) in sodium acetate buffer (0.1M, pH 4.7, chelex treated) and reacted in a glass vial for 60 min at 25 or 40°C (*n* = 3). The radiochemical yield of each reaction mixture was determined by instant thin-layer liquid chromatography (iTLC-SG, Varian, Diegem, Belgium). iTLC-SG papers were developed in an elution chamber using acetonitrile/water (75/25). The distribution of activity on the iTLC chromatograms was quantified using phosphor storage autoradiography [super-resolution screen, Perkin Elmer, Waltham, USA processed in a Cyclone Plus system (Perkin Elmer) and analyzed using Optiquant software (Perkin Elmer)].

Radiolabeling HSA-constructs: Purified HSA-constructs were labeled using 10 μM of the HSA-conjugate (90 μL) with [^161^Tb]TbCl_3_ (0.2 MBq, 10 μL, 50 mM HCl) at 40°C for 60 min (*n* = 3). Radiochemical yields were determined by iTLC-SG, eluted with sodium citrate buffer (0.1 M, pH 5.8). Radiolabeled HSA-constructs were additionally analyzed by radio-SEC using the method described above.

### *In vitro* Stability Studies

Stability of ligand complexes in phosphate buffered saline pH 7.4: The radiolabeled ligands were purified using a C18 Plus SEP-PAK cartridge (Waters, Antwerp, Belgium) by loading the reaction mixture, rinsing with water (5 mL) to remove unreacted [^161^Tb]TbCl_3_, and eluting the purified complex with abs. ethanol (0.5 mL). 80 μL of the ethanolic solution was added to 720 μL of sodium phosphate buffer (0.15 M sodium chloride, 0.01 M phosphate, pH 7.4, Thermo Fisher) and incubated at 37°C (*n* = 3). Samples were collected at different time points (10 min, 1 h, 4 h, and 24 h) and the percentage of intact ^161^Tb-complex was determined using the same iTLC chromatography system as used above.

Stability of HSA-ligand in human serum: After radiolabeling and without purification, 50 μL of the ^161^Tb-HSA radiolabeling solution was added to 720 μL human serum (Sigma Aldrich) and incubated at 37°C (*n* = 3). Samples were collected at different time points (10 min, 1 h, 4 h, and 24 h) and the percentage of intact ^161^Tb-HSA construct was determined using the same instant thin-layered liquid chromatography system as used in radiolabeling and referenced to the initial radiochemical yield. The *in vitro* stability was confirmed with the radio-SEC method described above at 1, 4 and 24 h.

Competition studies with EDTA: After radiolabeling and without purification, 50 μL of the ^161^Tb-HSA radiolabeling solution was added to 50 μL EDTA solution (10 mM, 0.1M PBS, pH 7.4, Sigma Aldrich) and incubated at 37°C (*n* = 3). Samples were collected at different time points after incubation (1 h, 4 h, and 24 h) and the percentage of intact ^161^Tb-HSA construct was determined using the same iTLC method mentioned above.

### Biodistribution Studies

Mice were anesthetized with 2.5% isoflurane in O_2_ at a flow of 1 L/min and injected with ~1 MBq of [^161^Tb]TbCl_3_ or ^161^Tb-HSA construct (0.1–0.3 nmoles) via a tail vein. Animals were sacrificed by decapitation at 10 min, 1 h, 4 h, 24 h, or 7 days post injection (*n* = 3 animals per time point). Blood and organs were collected in tubes, weighed, and radioactivity was determined using an automated gamma counter as described above. Results are presented as standardized uptake values [SUV; determined using SUV = (MBq_tissue_/g_tissue_)/(MBq_injected_/g_mouse_)]. For calculation of percentage injected dose (%ID) in blood, bone and muscle, masses were assumed to be 7, 12, and 40% of mouse body weight, respectively (23, 24). Blood data points (%ID_calculated_) were fitted to a standard half-life equation (least-squares regression analysis), $\%ID_{calc}=\;A\cdot 0.5^{k\Delta t}$, where A = constant, Δt = hours after injection (h), and k = 1/plasma half-life (h^−1^).

### Statistical Analysis

Quantitative data are expressed as mean ± SD unless stated otherwise. Means were compared using a mixed model ANOVA analysis in GraphPad Prism 9.1.2. Values were determined to be statistically significant for *p*-values less than the threshold value of 0.05.

## Results

### Optimization of Radiolabeling Conditions for Low Temperature Labeling

**Figure 2:**
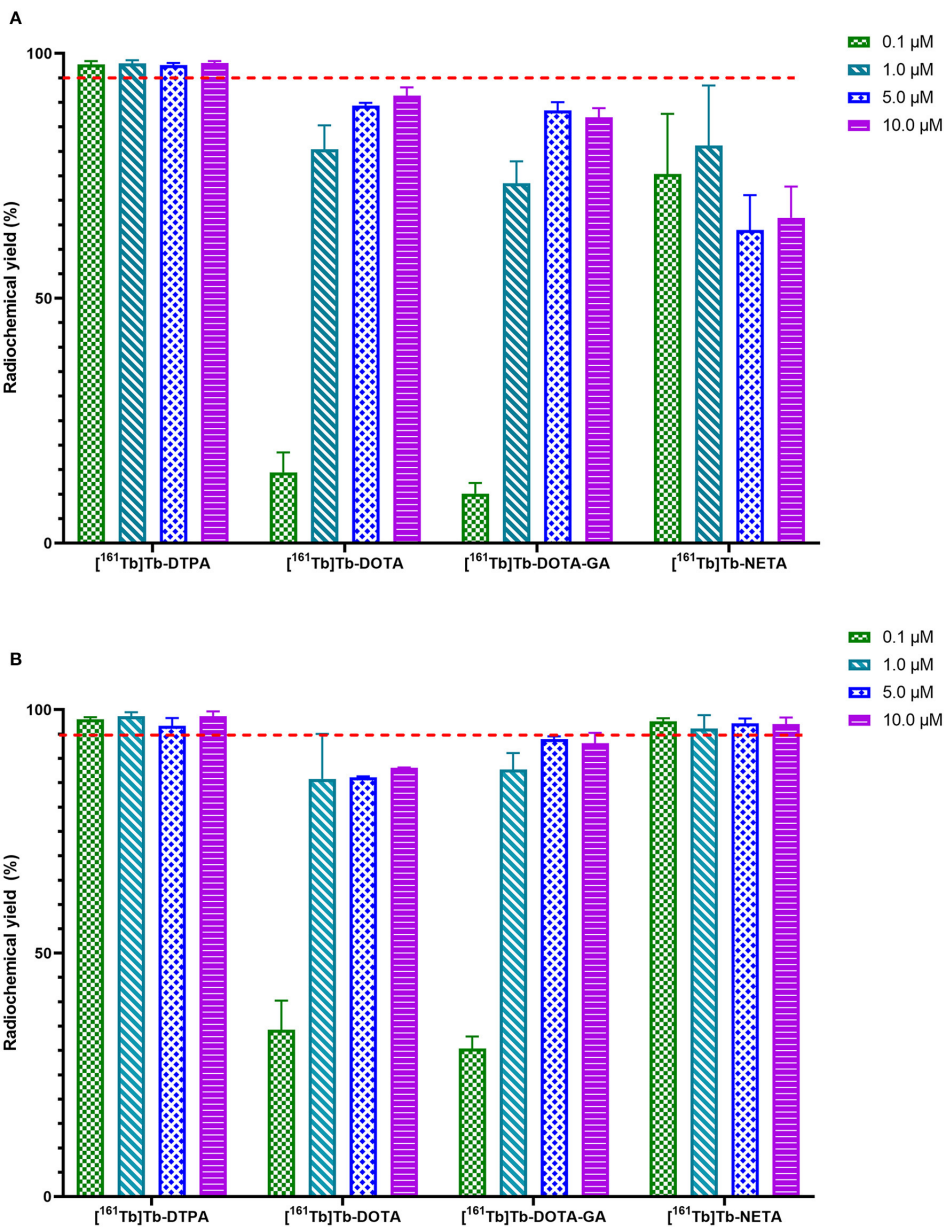
Radiochemical yields of [^161^Tb]Tb-**L** (where **L** = **DTPA**, **DOTA**, **DOTA-GA** or **NETA**) at 25°C **(A)** and 40°C **(B)** after 60 min. Red line is inserted to indicate quantitative yield (95%).

Radiolabeling efficiency of all ligands (**DTPA**, **DOTA**, **DOTA-GA**, **NETA**) was evaluated using four different ligand concentrations (0.1, 1, 5 and 10 μM) at 25°C and 40°C after 60 min reaction time. Results of the radiolabeling can be found in Figures 2A,B. Radiolabeling with **DTPA** resulted in >98% radiolabeling efficiency at all tested concentrations, even at 25°C. All other ligands required a temperature of 40°C to efficiently (>90%) chelate the terbium (III) ion. Radiolabeling using **NETA** at 40°C resulted in quantitative yields in all investigated ligand concentrations (labeling efficiency >97%). Both **DOTA** and **DOTA-GA** required higher concentrations to reach suffucient radiolabeling efficiency.

### *In vitro* Stability of Radiolabeled Ligands

**Figure 3:**
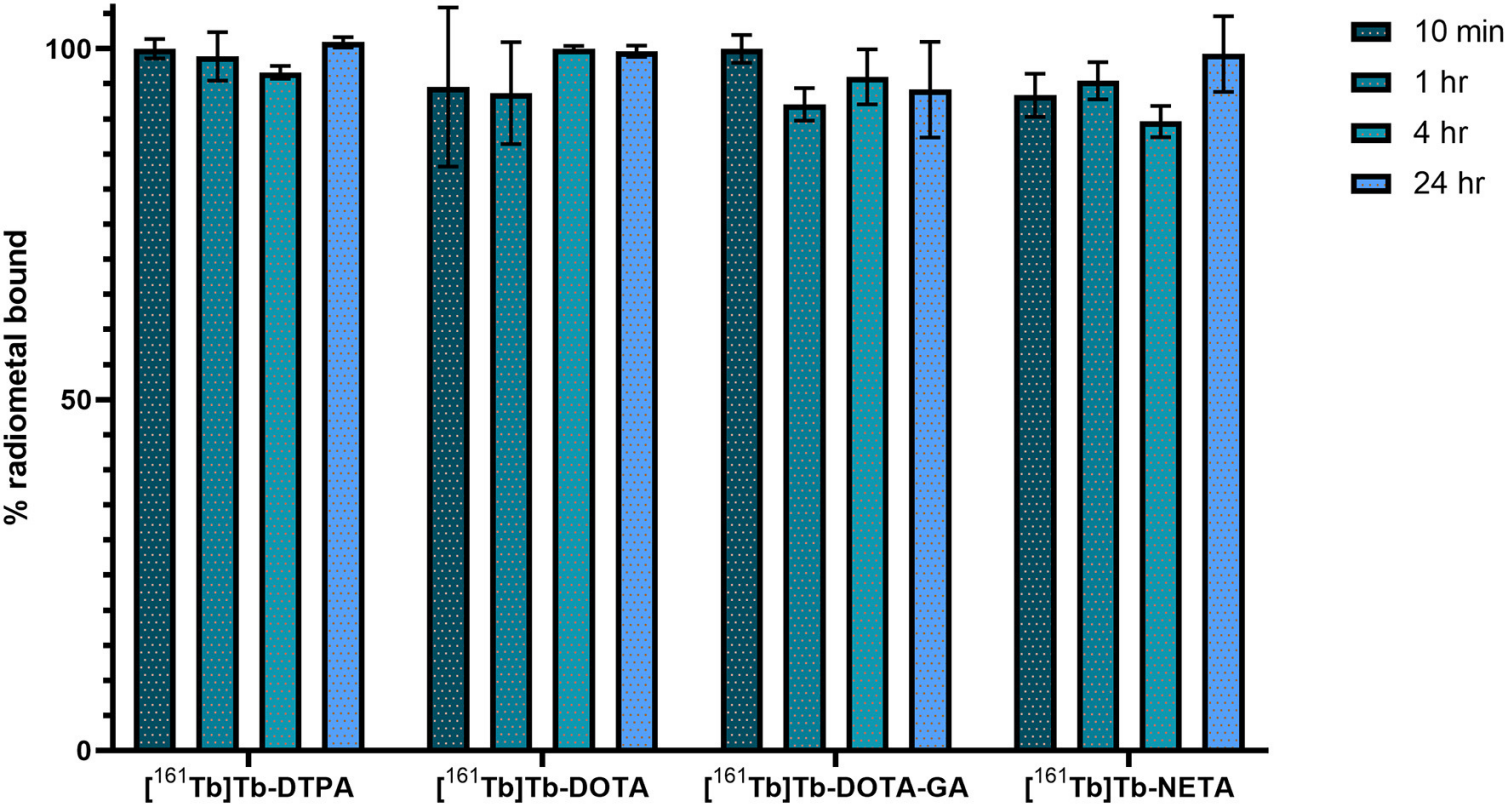
*In vitro* stability of ^161^Tb-radiolabeled complexes in PBS at 37°C.

Metal-ligand *in vitro* stability was determined in phosphate buffered saline (PBS, pH 7.4) at 37°C and analyzed over 24 h (Figure 3). The amount of ^161^Tb bound to the ligand was referenced to the initial radiochemical purity. For **DTPA**, **DOTA** and **NETA** ligand systems, >95% of the metal was still chelated to the ligand after 24 h. The **DOTA-GA** ligand was observed to have retained only 92.1 ± 6.8% of the initial radiochemical purity over the same period.

### Synthesis, Radiolabeling and *in vitro* Stability of Human Serum Albumin Conjugates

**DTPA**, **DOTA**, **DOTA-GA** or **NETA** was reacted with HSA in a 5:1 molar excess. HSA-constructs were purified using a size exclusion cartridge and analyzed using HPLC-SEC. Unconjugated human serum albumin was found to be retained in the size exclusion column for 19 min and HSA-chelator constructs eluted at the same retention time (Supplementary Figures 1–6). Constructs were analyzed using mass spectrometry to determine the number of ligands attached to the HSA protein. An increase of 1,000–1,300 Da was observed for each conjugate, indicating an average number of two chelators per albumin molecule. The mass spectra data is summarized in Table 1.

**Table 1:** Mass spectrum data of HSA and compounds L-HAS.

**Compound**	**Mass found (kDa)**	**Chelators per albumin**
HSA	66.478	N/A
HSA-DTPA	67.852	~2
HSA-DOTA	67.688	~2
HSA-DOTA-GA	67.821	~2
HSA-NETA	67.726	~2

**Figure 4:**
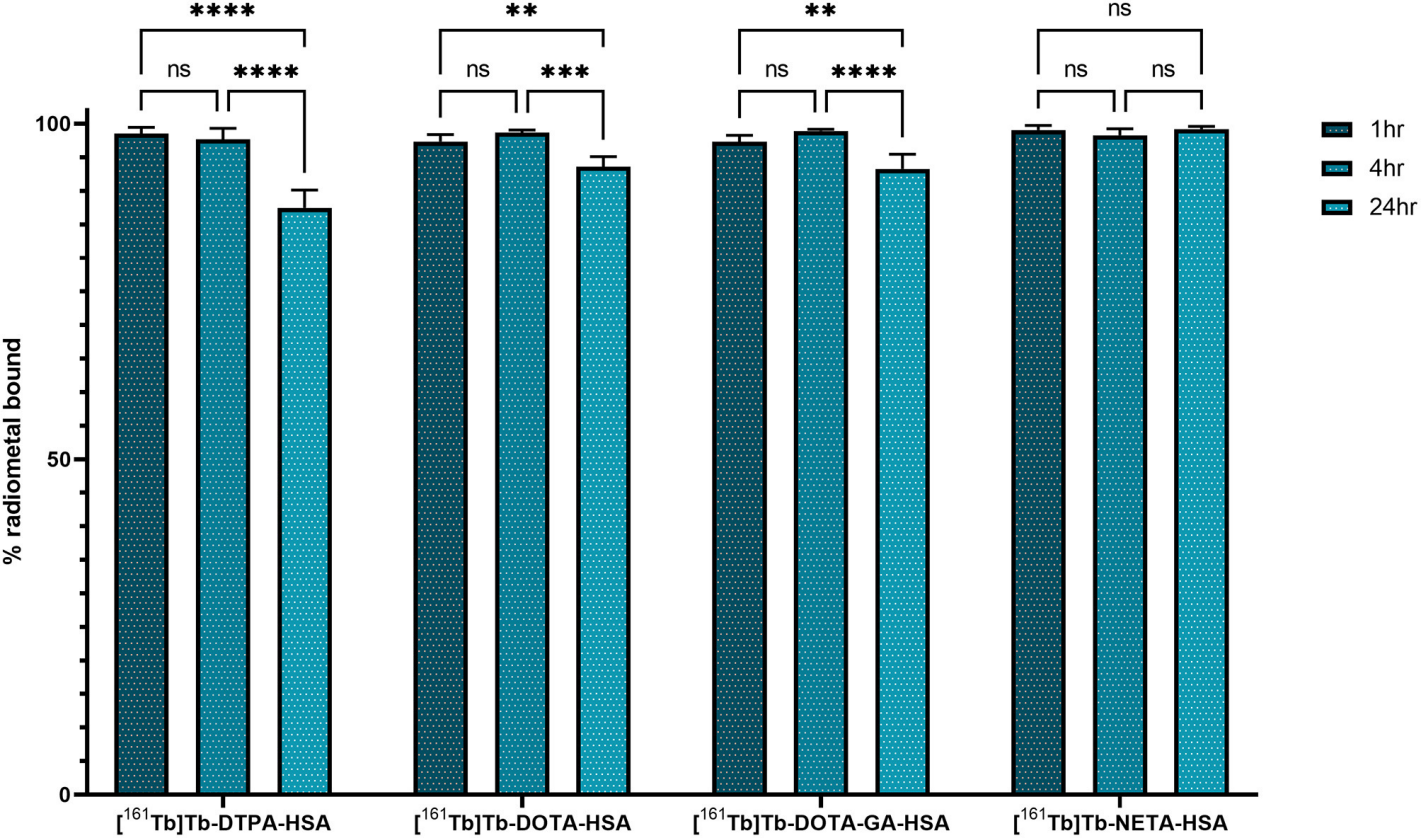
*In vitro* stability of ^161^Tb-radiolabeled HSA-complexes in human serum at 37°C. Significant values are expressed as *P* < ^*^0.05, ^**^0.01, ^***^0.001, ^****^0.0001.

HSA-conjugates and unconjugated HSA were radiolabeled at a concentration of 10 μM with [^161^Tb]TbCl_3_ at 40°C to ensure maximum radiochemical yield. HSA (not conjugated to any chelator) only coordinated 6.0 ± 1.2% of the [^161^Tb]TbCl_3_ in the reaction mixture. The investigated conjugates were all labeled with quantitative yields (>98%) (Supplementary Table 1) as determined with iTLC and radio-SEC-HPLC (Supplementary Figures 3–6). The radiolabeled constructs (without further purification) were added to human serum and incubated at 37°C for 24 h. [^161^Tb]Tb-**DOTA**-HSA, [^161^Tb]Tb-**DOTA-GA**-HSA and [^161^Tb]Tb-**NETA**-HSA remained intact (>93%) for at least 24 h. In contrast, only 87.5 ± 2.6% of radiometal remained coordinated to [^161^Tb]Tb-**DTPA**-HSA after 24 h (Figure 4). Radio-SEC-HPLC chromatograms of ^161^Tb-labeled HSA conjugates are provided in the supporting information (Supplementary Figures 7–10). To assess the susceptibility of trans-chelation, the labeled HSA-conjugates were incubated with 1,000-fold excess of EDTA (Supplementary Figure 11). As observed in the previous study, ^161^Tb leached from the **DTPA**-HSA ligand system, with only 64.4 ± 0.9% of the initial ^161^Tb remained bound to HSA after 24 h at 37°C. For the other conjugates, >90% of the initial fraction of the radiometal remained bound to HSA after 24 h.

### *Ex vivo* Biodistribution of [^161^Tb]TbCl_3_ and ^161^Tb-Labeled HSA-Conjugates

**Figure 5:**
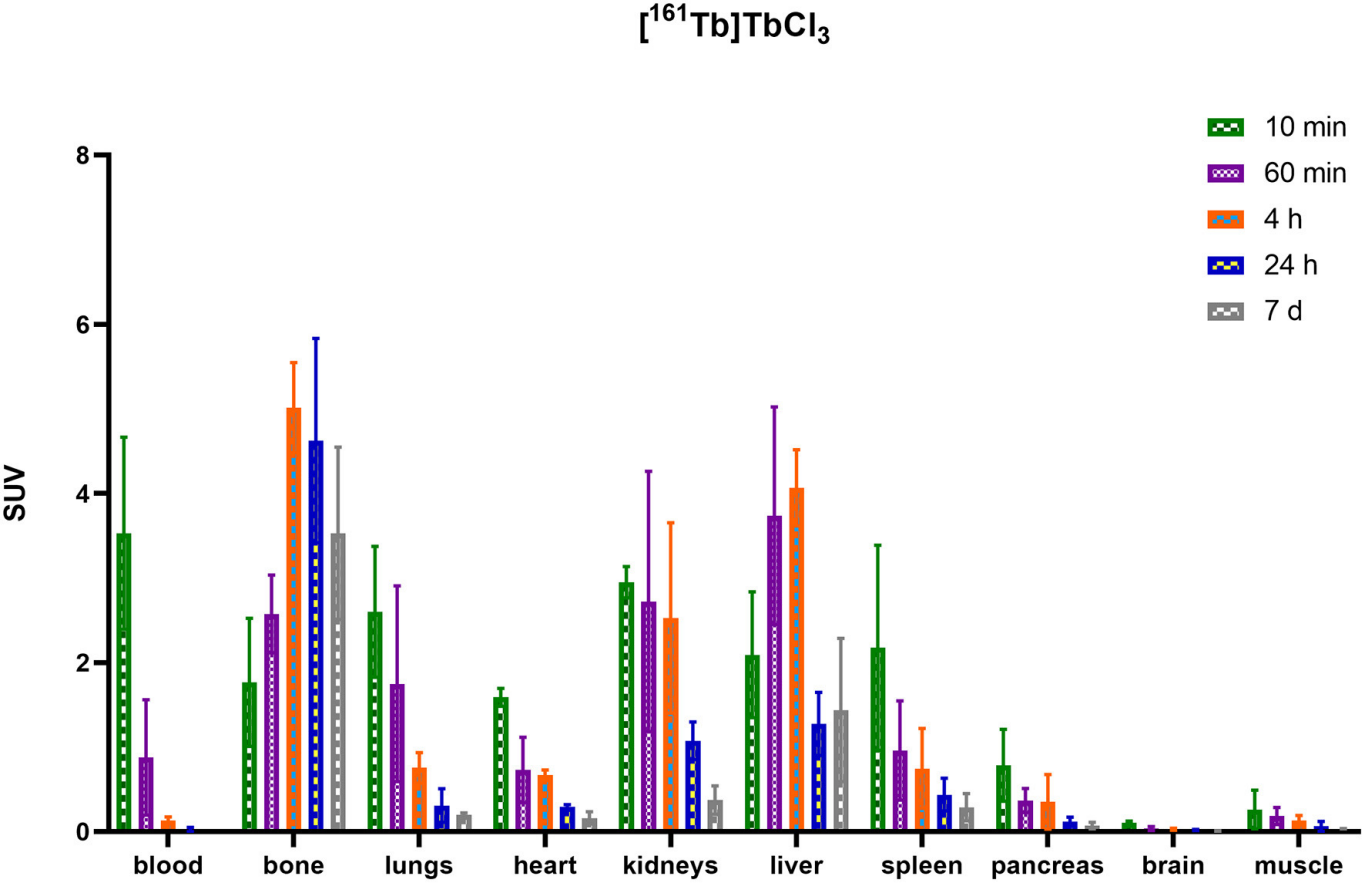
Biodistribution of [^161^Tb]TbCl_3_ expressed in standardized uptake values per organ.

**Figure 6:**
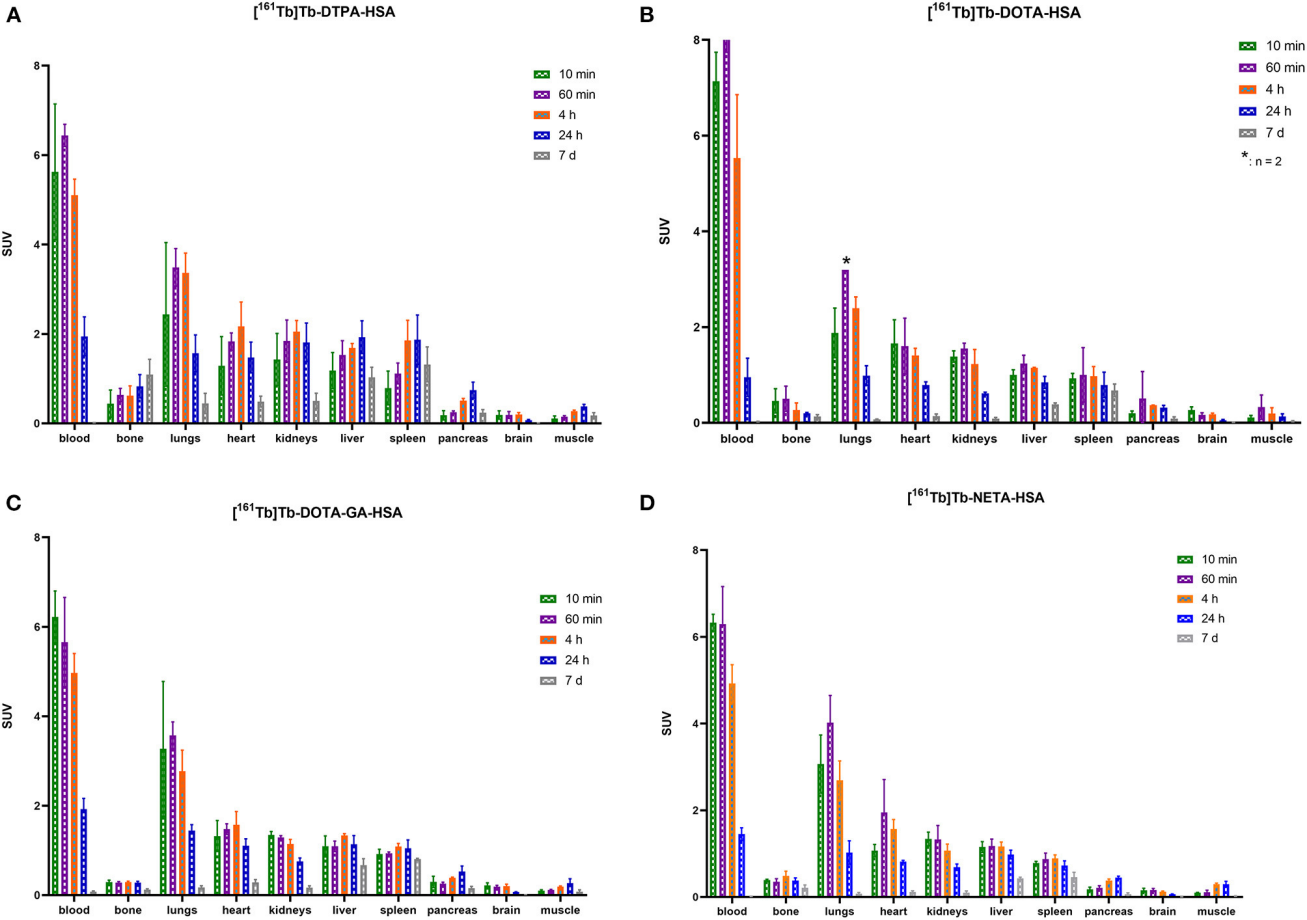
Biodistribution of [^161^Tb]Tb-**DTPA**-HSA **(A)**, [^161^Tb]Tb-**DOTA**-HSA **(B)**, [^161^Tb]Tb-**DOTA-GA**-HSA **(C)** and [^161^Tb]Tb-**NETA**-HSA **(D)** expressed in standardized uptake values per organ (*n = 2).

HSA-conjugates were labeled with [^161^Tb]TbCl_3_ and injected into mice intravenously (tail vein). Additionally, [^161^Tb]TbCl_3_ (PBS, pH 7.4) was injected in a control group to identify the organs in which ^161^Tb accumulates in case it leaches from the complex. Unconjugated [^161^Tb]TbCl_3_ showed high accumulation in bone and liver (Figure 5; Table 2; Supplementary Tables 4–6). Four hours after injection of [^161^Tb]TbCl_3_, an SUV of 5.0 ± 0.5 (60.2 ± 6.4% ID) and 4.1 ± 0.4 (23.8 ± 2.5% ID) was observed for bone and liver, respectively. A bone-to-blood ratio of 4.8 ± 3.8 was observed already after 1 h post injection (p.i.) and this value further increased reaching a bone-to-blood ratio of 39.3 ± 13.0 after 4 h p.i. (Supplementary Table 2). The half-life of the free [^161^Tb]TbCl_3_ in blood was about 0.4 h (Supplementary Figure 17). As could be expected, the HSA constructs did not show any specific accumulation in any target tissue. No significant bone uptake of activity was observed for the [^161^Tb]Tb-**DOTA**-HSA, [^161^Tb]Tb-**NETA**-HSA, and [^161^Tb]Tb-**DOTA-GA**-HSA conjugates (Figures 6A–D, Table 2). In contrast, in the mice injected with [^161^Tb]Tb-**DTPA**-HSA, increasing bone uptake was observed (SUV: 0.8 ± 0.3 and 1.1 ± 0.3 at 24 h and 7 days p.i., respectively) in function of time (Table 2), suggesting *in vivo* dissociation and absorption of free ^161^Tb in bone. The blood half-life of the HSA constructs was significantly longer than for [^161^Tb]TbCl_3_ (8–15 h, Supplementary Figures 17–21). Standardized uptake value graphs are provided in Figure 6, with % injected activity diagrams provided in Supplementary Figures 12–16. %ID, %ID/g and SUV values are presented in Supplementary Tables 4–18.

**Table 2:** SUV values of bone uptake in healthy mice.

	**[^**161**^Tb]TbCl_**3**_**	**[^**161**^Tb]Tb -DTPA-HSA**	**[^**161**^Tb]Tb -DOTA-HSA**	**[^**161**^Tb]Tb -DOTA-GA-HSA**	**[^**161**^Tb]Tb -NETA-HSA**
10 min	1.77 ± 0.76	0.44 ± 0.30	0.46 ± 0.25	0.29 ± 0.05	0.39 ± 0.02
1 h	2.58 ± 0.46	0.64 ± 0.15	0.50 ± 0.26	0.28 ± 0.02	0.35 ± 0.07
4 h	5.02 ± 0.53	0.62 ± 0.22	0.27 ± 0.15	0.29 ± 0.02	0.49 ± 0.11
24 h	4.63 ± 1.21	0.83 ± 0.26	0.20 ± 0.01	0.28 ± 0.03	0.38 ± 0.06
7 d	3.53 ± 1.02	1.1 ± 0.34	0.13 ± 0.04	0.12 ± 0.02	0.21 ± 0.06

## Discussion

In this study, we aimed to develop techniques that allow radiolabeling of heat sensitive biomolecules with ^161^Tb. A series of ligands were preselected based on their lanthanide chelating capacity reported in literature (20, 25, 26). For each ligand, we evaluated the effect of ligand concentration and temperature on radiolabeling yields. Finally, stability of the different ligand complexes was evaluated *in vitro* and *in vivo*.

Coordination of terbium is pH sensitive, as too low pH blocks carboxyl coordination, which is the main coordinating moiety of the ligands of interest (Supplementary Table 3). In aqueous terbium solutions, hydrolysis (formation of Tb(H_2_O)_x_(OH)_y_ species) will occur at increased pH (pH ~ 6–7.6) (3, 10, 12, 27–29). Hydrolysis will dramatically reduce or prevent the overall formation of Tb-ligand complex, which might be mitigated by increasing the temperature during radiolabeling. We selected low pH conditions [sodium acetate buffer (0.1 M, pH 4.7)] to prevent hydrolysis and enable low-temperature chelation of Tb. When radiolabeling the **DTPA** ligand, quantitative radiochemical yields (>98%) were observed for all conditions investigated. Incubating the reaction mixture at 25°C was enough to obtain quantitative yields, even at low ligand concentrations (Figure 2A). This can be attributed to the flexible nature of the linear DTPA framework which makes chelating the terbium (III) ion easier (1). At 25°C, radiochemical yields of **DOTA** and **DOTA-GA** were lower, with a maximum radiochemical yield of 91% (10 μM). This could be expected in view of the more rigid tetraaza ring of the latter two ligand structures. Increasing the temperature to 40°C yielded no change in the maximum yields obtained for higher concentrations of **DOTA** and **DOTA-GA** but allowed for better radiochemical yields in the low concentrations tested (Figure 2B). Finally, for **NETA**, a mean radiochemical yield of ~60% was observed at 25°C but quantitative yields (>95%), comparable to **DTPA**, were obtained at 40°C. The hybrid nature of the **NETA** framework could explain the radiochemical yields similar to **DOTA** and **DOTA-GA** at 25°C. Slightly increasing the temperature however seems to provide enough energy to allow terbium(III) to be incorporated more efficiently into the chelator binding pocket.

The stability of the ^161^Tb-ligand bond was evaluated in a phosphate buffered saline solution (pH 7.4) at 37°C during a time period of 24 h using instant thin-layer chromatography (Figure 3). At the end of the study, >95% (relative to the initial radiochemical purity) of the metal remained intact for complexes with **DTPA**, **DOTA** and **NETA**. The complex with **DOTA-GA** was found to be the least stable, with 92.1 ± 6.8% of the initially chelated metal intact after 24 h.

After optimizing the radiolabeling conditions, we used these optimized conditions to radiolabel HSA conjugates, as a proof of concept. HSA is a heat sensitive molecule and is the most abundant protein in blood essential for the transport of many proteins throughout the body (30, 31). It has a prolonged serum half-life (30), which also makes it advantageous for determining long-term *in vivo* stability of radiolabeled conjugates. Additionally, since HSA circulates in the blood and shows minimal physiological accumulation in tissue, it is the perfect tool to evaluate dissociation and potential accumulation of the free radiometal to other tissues. Bifunctional ligands were conjugated to HSA non-regioselectively, using lysine coupling. The ligands were reacted with HSA to afford conjugates **L**-HSA (where **L** = **DTPA**, **DOTA**, **DOTA-GA** or **NETA**), and analyzed by UV-HPLC and high-resolution mass spectrometry. Only a single peak (Rt = 19 min) was recorded in the UV channel (**L**-HSA), and their retention time is identical to that of underivatized HSA (Supplementary Figures 1, 3–6). No aggregation or degradation products were observed via SEC-HPLC. Furthermore, high resolution mass spectrometry was used to estimate the number of ligands conjugated to HSA for every conjugate. Unconjugated HSA was used as a reference for calculating the number of ligand molecules that are conjugated (66477- 66485 Da) to HSA. The molecular mass of all the conjugates increased by 1,000–1,300 Da relative to HSA, which suggests that the conjugates have an average of two ligands per HSA moiety (Table 1).

Using the optimized labeling conditions (60 min, 40°C), HSA constructs **L**-HSA were radiolabeled with ^161^Tb and the labeling reaction mixture was analyzed by iTLC and radio-SEC. In addition, non-derivatized HSA was incubated with [^161^Tb]TbCl_3_ to determine if there is any non-chelator related binding of terbium to the protein. The ^161^Tb-labeled conjugates ([^161^Tb]Tb-**L**-HSA) were found to have radiochemical purity >98% for all constructs. In contrast, unconjugated HSA was observed to have a radiochemical purity of only 6.0 ± 1.2%, suggesting minimal complexation of ^161^Tb occurs in absence of a chelator (Supplementary Table 1). RadioHPLC analysis of the HSA-constructs confirms successful coordination of ^161^Tb to the investigated HSA conjugates (Supplementary Figures 1–6).

Upon incubation in human serum, a radiochemical purity above 95% was maintained for the radiolabeled HSA constructs [^161^Tb]Tb-**DOTA**-HSA, [^161^Tb]Tb-**DOTA-GA**-HSA and [^161^Tb]Tb-**NETA**-HSA over a 24-h study period. [^161^Tb]Tb-**DTPA**-HSA had a noticeable decrease in radiochemical purity after 24 h from 98.6 ± 0.5% to 88.1 ± 1.3%. This is commonly observed with ligands bearing the DTPA chelating framework, as it is often labeled as an “easy-in-easy-out" ligand for metals (18). In a competition study with EDTA (1,000× molar excess), [^161^Tb]Tb-**DOTA**-HSA, [^161^Tb]Tb-**DOTA-GA**-HSA and [^161^Tb]Tb-**NETA**-HSA was observed to show minimal transchelation of ^161^Tb, with >90% of metal still associated to L-HSA. In stark contrast, >35% of ^161^Tb transchelated from **DTPA**-HSA to EDTA (Supplementary Figure 11). These *in vitro* results indicate that **DTPA** is a poor choice for chelation of terbium.

As described before, HSA can be used as an effective model protein to evaluate the *in vivo* stability of radiolabeled complexes (30). First, a biodistribution was performed with [^161^Tb]TbCl_3_ to determine its *in vivo* fate. Free [^161^Tb]TbCl_3_ was observed to clear from the blood within the first 24 h (Figure 5; Supplementary Tables 4–6) and high uptake and retention were observed in liver and bone, with the highest values observed at 4 h p.i (SUV_liver_ = 4.1 ± 0.4 and SUV_bone_ 5.0 ± 0.5). At day 7, still high retention of radioactivity in bone and liver was observed (SUV_liver_ = 1.4 ± 0.8, SUV_bone_ = 3.5 ± 1.0), resulting in a strongly increasing bone-to-blood and bone-to-muscle ratio over the 7-day period (Supplementary Table 2). The high accumulation of radioactivity in bone, allowed us to identify this tissue as an indicator for leaching of the radionuclide from the radiopharmaceutical *in vivo*. The biodistribution of the ^161^Tb-labeled HSA constructs showed the expected accumulation of activity in organs with high blood content (heart, lungs, spleen, etc.). As observed with the free [^161^Tb]TbCl_3_, increased bone and liver uptake was observed over the entire 7-day period for [^161^Tb]Tb-**DTPA**-HSA (Figure 6A). At 7 days post injection of [^161^Tb]Tb-**DTPA**-HSA, a SUV_bone_ value of 1.1 ± 0.3 was observed. This is significantly higher (*P* < 0.001) than for the other three constructs ([^161^Tb]Tb-**DOTA**-HSA: SUV_bone_ 0.1 ± 0.0; [^161^Tb]Tb-**DOTA-GA**-HSA: SUV_bone_ 0.1 ± 0.0; [^161^Tb]Tb-**NETA**-HSA: SUV_bone_ 0.2 ± 0.1), strongly suggesting that there is *in vivo* dissociation of the metal from the **DTPA**-HSA ligand (Figures 6B–D). This result, together with the *in vitro* stability data in human serum and EDTA competition study, strongly suggests **DTPA** has fast radiolabeling kinetics but does not adequately retain the radiometal after chelation. In the *in vitro* test with human serum, 10% of the radioactivity of [^161^Tb]Tb-**DTPA**-HSA was dissociated after 24 h. After 24 h *in vivo* studies showed 10% of the injected activity in the bone (Supplementary Figure 12), showing a good concordance between *in vitro* and *in vivo* results. No increase in retention of liver and bone activity was observed over 7 days after injection of radiolabeled constructs with **DOTA**-HSA, **DOTA-GA**-HSA and **NETA**-HSA (Figures 6B–D), suggesting high *in vivo* stability of ^161^Tb complexes with ligands **DOTA**, **DOTA-GA** and **NETA** as compared to **DTPA**. This is an important result for further studies with radioactive terbium isotopes as the CHX-A”-DTPA framework (**DTPA**, Figure 1) is often seen and used as a generic chelator for different radiometals (32). **DOTA**, **DOTA-GA** and **NETA** have more rigid frameworks, which can explain the more stable chelation of metals *in vivo*.

As expected, radiolabeled HSA-constructs were retained longer in blood compared to [^161^Tb]TbCl_3_. The blood biological half-life increased from 0.4 h (free [^161^Tb]TbCl_3_, Supplementary Figure 17) to 8–14 h ([^161^Tb]Tb-**DTPA**-HSA: 14.8 h; [^161^Tb]Tb-**DOTA**-HSA: 8.6 h; [^161^Tb]Tb-**DOTA-GA**-HSA: 14.2 h; [^161^Tb]Tb-**NETA**-HSA: 10.8 h; Supplementary Figures 18–21). This minor variation in blood biological half-life of the different conjugates might be attributed to the non-regioselective coupling of ligands to HSA; potentially reacting with regions essential to biological circulating proteins (neonatal Fc receptor, etc.). Therefore, in future experiments, it is essential to make use of more site-specific targeting approaches (his-tag coupling, *sortase A*, etc.) (33–35) to avoid interfering with the binding affinity of the biomolecule.

## Conclusion

This study is the first report on labeling with ^161^Tb in mild conditions (25°C and 40°C in aqueous buffer). As a proof of concept, we successfully radiolabeled the heat-sensitive biomolecule HSA with ^161^Tb, with high radiochemical yields. Several bifunctional ligands were evaluated for their radiolabeling properties, as well as their *in vivo* and *in vitro* stability. Of these ligands, radiolabeling with **DTPA** was highly efficient, even at room temperature. However, the **DTPA**-HSA construct showed the lowest stability, both *in vitro* and *in vivo*, leading to significant off-target bone uptake and retention. In contrast, complexes with a more rigid backbone (**DOTA**, **DOTA-GA** and **NETA**) required slightly higher radiolabeling temperatures but were found to be very stable *in vitro* and *in vivo*. These ligands have potential to be used with other vector molecules for diagnostic and therapeutic applications of the terbium radioisotope family. Research is currently ongoing to conjugate these ligands to other heat-sensitive vector molecules to allow delivery of ^161^Tb or other terbium radioisotopes to the biological target of interest.

## Data Availability Statement

The original contributions presented in the study are included in the article/Supplementary Material, further inquiries can be directed to the corresponding author/s.

## Ethics Statement

The animal study was reviewed and approved by Ethical Committee for Animal Experimentation, KU Leuven.

## Author Contributions

Experimental work was performed by IC and SA. IC, GB, TC, MO, and FC designed this research. IC, MO, and FC analyzed the data. [^161^Tb]TbCl_3_ was produced and purified by AB and MV. IC, MO, and FC drafted the manuscript. All authors contributed to the article and approved the submitted version.

## Conflict of Interest

The authors declare that the research was conducted in the absence of any commercial or financial relationships that could be construed as a potential conflict of interest.

## Publisher's Note

All claims expressed in this article are solely those of the authors and do not necessarily represent those of their affiliated organizations, or those of the publisher, the editors and the reviewers. Any product that may be evaluated in this article, or claim that may be made by its manufacturer, is not guaranteed or endorsed by the publisher.

## Abbreviations

HSA: human serum albumin
SEC: size exclusion chromatography
HPLC: high-performance liquid chromatography
iTLC: instant thin-layered liquid chromatography
SUV: standardized uptake value.
