# Supplementary material for: Radiolabeling of Human Serum Albumin With Terbium-161 Using Mild Conditions and Evaluation of in vivo Stability
Source: Front Med (Lausanne). 2021 Aug 18;8:675122. doi: 10.3389/fmed.2021.675122 (PMC8422959; doi:10.3389/fmed.2021.675122)
Supplement: Supplementary file 1 [file Data_Sheet_1.docx]

Supporting Information

Radiolabeling of human serum albumin with terbium-161 using mild conditions and evaluation of *in vivo* stability

Irwin Cassells ^1,2^, Stephen Ahenkorah ^1,2^, Andrew R. Burgoyne ^2^, Michiel Van de Voorde ^2^, Christophe M. Deroose ^3^, Thomas Cardinaels ^2,4^, Guy Bormans ^1^, Maarten Ooms ^2*^ and Frederik Cleeren ^1*^

^1^ Radiopharmaceutical Research, Department of Pharmacy and Pharmacology, KU Leuven, Leuven, Belgium

^2^ Institute for Nuclear Materials Science, Belgian Nuclear Research Centre (SCK CEN), Mol, Belgium

^3^ Nuclear Medicine, University Hospital Leuven & Nuclear Medicine and Molecular Imaging, Department of Imaging and Pathology, KU Leuven, Leuven, Belgium

^4^ Department of Chemistry, KU Leuven, Heverlee, Belgium

*** Correspondence:**Maarten Ooms, maarten.ooms@sckcen.be
Frederik Cleeren, frederik.cleeren@kuleuven.be

Keywords: Terbium-161, radiopharmaceutical, radiolabeling, TRNT, bio-conjugation.

**Figure S1:** Chromatogram (280 nm) of human serum albumin (HSA), eluted on a Superdex 200 10/300 GL with PBS at 0.75 mL/min.


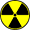


**Figure S2:** Radio-SEC of [^161^Tb]TbCl_3_, eluted on a Superdex 200 10/300 GL with PBS at 0.75 mL/min. Radioactive channel with peak window 50-200 keV.


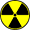


**Figure S3:** UV spectrum (280 nm) of [^161^Tb]Tb-**DTPA**-HSA and radioactive signals, eluted on a Superdex 200 10/300 GL with PBS at 0.75 mL/min. Top: UV spectrum at 280 nM; Bottom: Radioactive channel with peak window 50-200 keV.


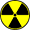


**Figure S4:** UV spectrum (280 nm) of [^161^Tb]Tb-**DOTA**-HSA and radioactive signals, eluted on a Superdex 200 10/300 GL with PBS at 0.75 mL/min. Top: UV spectrum at 280 nM; Bottom: Radioactive channel with peak window 50-200 keV.


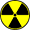


**Figure S5:** UV spectrum (280 nm) of [^161^Tb]Tb-**DOTA-GA**-HSA and radioactive signals, eluted on a Superdex 200 10/300 GL with PBS at 0.75 mL/min. Top: UV spectrum at 280 nM; Bottom: Radioactive channel with peak window 50-200 keV.


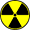


**Figure S6:** UV spectrum (280 nm) of [^161^Tb]Tb-**NETA**-HSA and radioactive signal, eluded on a Superdex 200 10/300 GL with PBS at 0.75 mL/min. Top: UV spectrum at 280 nM; Bottom: Radioactive channel with peak window 50-200 keV.


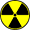

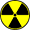

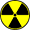

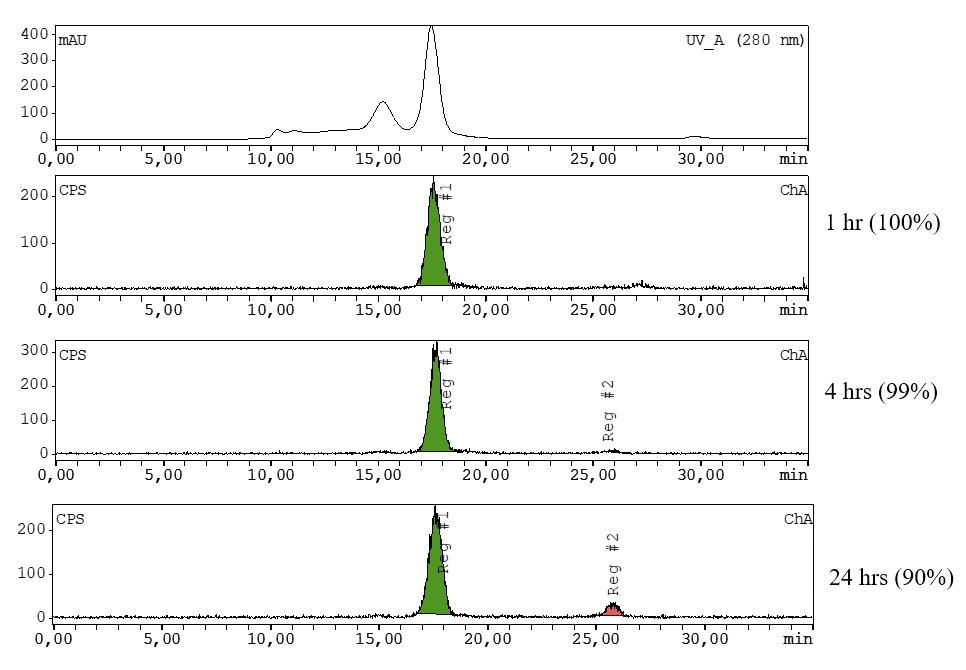


**Figure S7:** UV spectrum (280 nm) of [^161^Tb]Tb-**DTPA**-HSA in human serum and radioactive signal after 1, 4 and 24 hrs in human serum at 37 °C, eluded on a Superdex 200 10/300 GL with PBS at 0.75 mL/min. % radiometal bound is indicated in brackets.


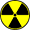

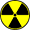

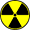

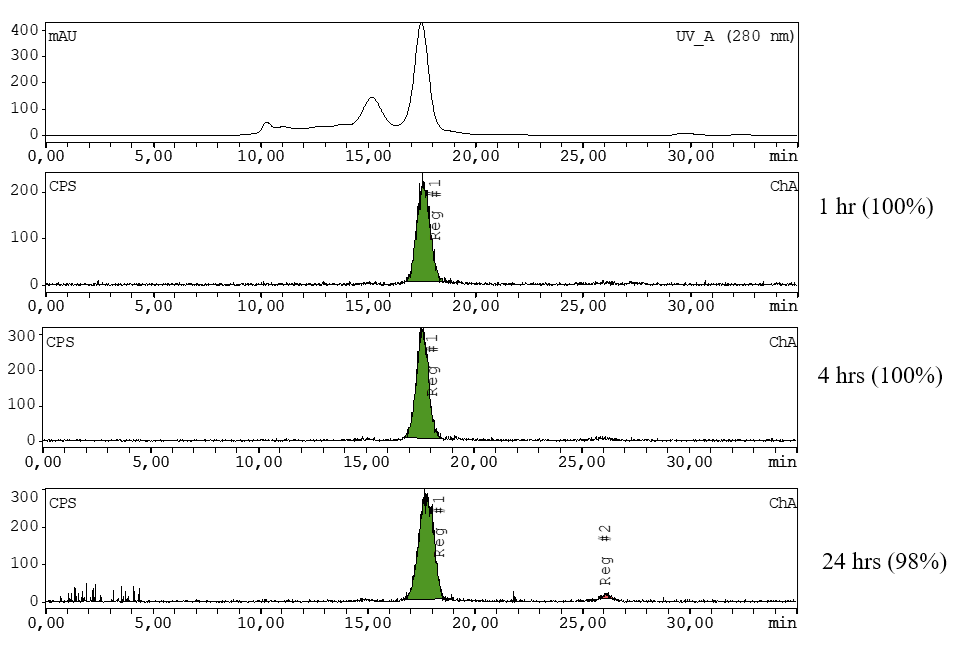


**Figure S8:** UV spectrum (280 nm) of [^161^Tb]Tb-**DOTA**-HSA in human serum and radioactive signal after 1, 4 and 24 hrs in human serum at 37 °C, eluded on a Superdex 200 10/300 GL with PBS at 0.75 mL/min. % radiometal bound is indicated in brackets.


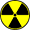

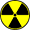

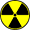

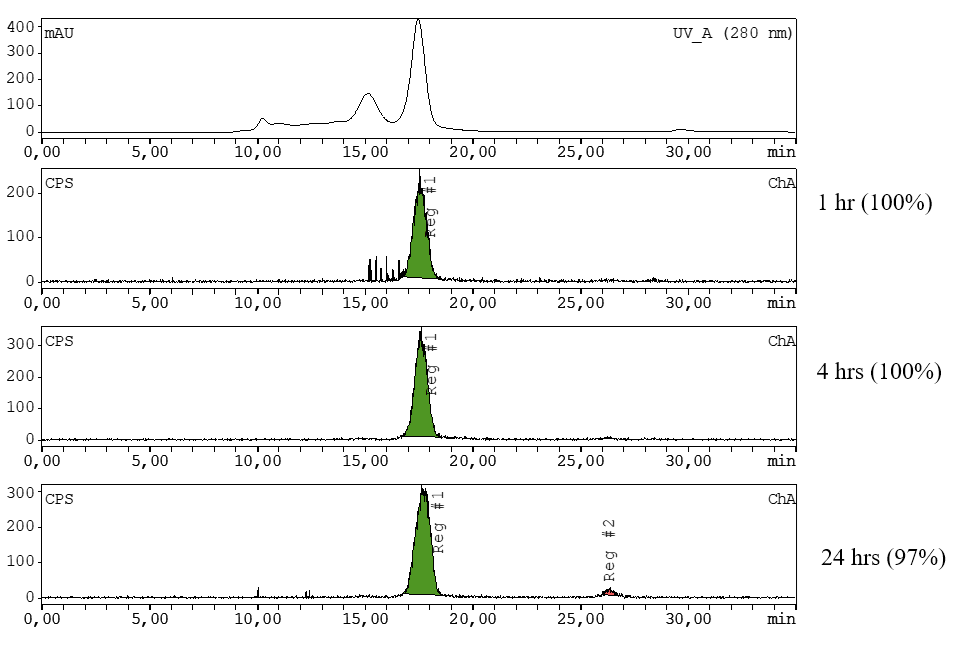


**Figure S9:** UV spectrum (280 nm) of [^161^Tb]Tb-**DOTA-GA**-HSA in human serum and radioactive signal after 1, 4 and 24 hrs in human serum at 37 °C, eluded on a Superdex 200 10/300 GL with PBS at 0.75 mL/min. % radiometal bound is indicated in brackets.


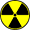

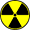

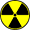

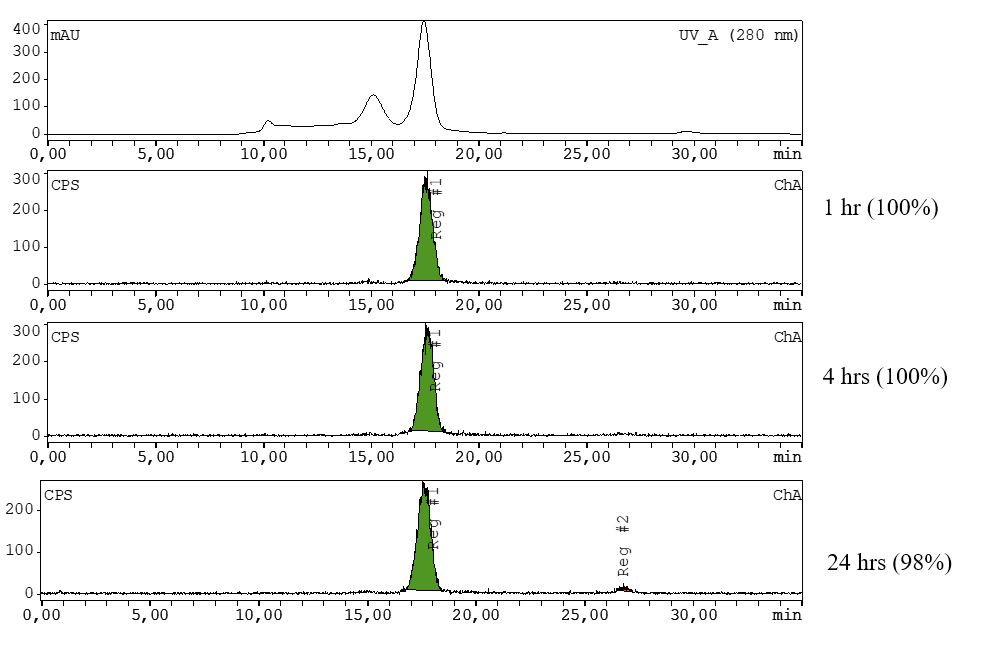


**Figure S10:** UV spectrum (280 nm) of [^161^Tb]Tb-**NETA**-HSA in human serum and radioactive signal after 1, 4 and 24 hrs in human serum at 37 °C, eluded on a Superdex 200 10/300 GL with PBS at 0.75 mL/min. % radiometal bound is indicated in brackets.

**Figure S11:** % radiometal bound after incubating with EDTA (100-fold excess) at 37 °C. Samples were taken after 1, 4 and 24 hours. Significant values are expressed as *P* < * = 0.05, ** = 0.01, *** = 0.001, **** = 0.0001.

**Figure S12:** Biodistribution of [^161^Tb]TbCl_3_ expressed in percentage of injected activity.

**Figure S13:** Biodistribution of [^161^Tb]Tb-**DTPA**-HSA expressed in percentage of injected activity.

**Figure S14:** Biodistribution of [^161^Tb]Tb-**DOTA**-HSA expressed in percentage of injected activity.

**Figure S15:** Biodistribution of [^161^Tb]Tb-**DOTA-GA**-HSA expressed in percentage of injected activity.

**Figure S16:** Biodistribution of [^161^Tb]Tb-**NETA**-HSA expressed in percentage of injected activity.

## Serum half-life determination (Figure S17-S21)

Percentage of injected activity (%IA) for blood uptake and time (hours) were fitted to a decay exponential function (y(t) = A x 0.5 ^kt^) with Microsoft Office 365 data solver. Using the k-value, t_1/2_ was determined using t_1/2_ = 1/k.

R^2^ = 0.9976; k = 2.3970; t_1/2_ = 0.41 hours

**Figure S17:** Percentage of injected activity (%IA) per time point for experimental (*ex vivo* result) and theoretical (predicted values) for [^161^Tb]TbCl_3_.

R^2^ = 0.9812; k = 0.067699; t_1/2_ = 14.77 hours

**Figure S18:** Percentage of injected activity (%IA) per time point for experimental (*ex vivo* result) and theoretical (predicted values) for [^161^Tb]Tb-**DTPA**-HSA.

R^2^ = 0.9974; k = 0.116057; t_1/2_ = 8.62 hours

**Figure S19:** Percentage of injected activity (%IA) per time point for experimental (*ex vivo* result) and theoretical (predicted values) for [^161^Tb]Tb-**DOTA**-HSA.

R^2^ = 0.9978; k = 0.070451; t_1/2_ = 14.19 hours

**Figure S20:** Percentage of injected activity (%IA) per time point for experimental (*ex vivo* result) and theoretical (predicted values) for [^161^Tb]Tb-**DOTA-GA**-HSA.

R^2^ = 0.9966; k = 0.092273; t_1/2_ = 10.84 hours

**Figure S21:** Percentage of injected activity (%IA) per time point for experimental (*ex vivo* result) and theoretical (predicted values) for [^161^Tb]Tb-**NETA**-HSA.

**Table S1.** Radiochemical yields of HSA and HSA constructs with [^161^Tb]TbCl_3_ (n = 3)

| Ligand | Radiochemical yield (%) |
| --- | --- |
| DTPA-HSA | 98.3 ± 0.3 |
| DOTA-HSA | 99.2 ± 0.6 |
| DOTA-GA-HSA | 99.2 ± 0.7 |
| NETA-hSA | 99.7 ± 0.0 |
| HSA | 6.0 ± 1.2 |

**Table S2.** Bone-to-blood ratio (SUV_bone_/SUV_blood_) and bone-to-muscle ratio (SUV_bone_/SUV_muscle_) for the different labeled constructs over time. Values were compared to 10 min time point to determine if significantly different (*P* < * = 0.05, ** = 0.01, *** = 0.001, **** = 0.0001)

| Bone-to-blood | 10 min | 1 hr | 4 hr | 24 hr | 7 d |
| --- | --- | --- | --- | --- | --- |
| ^161^TbCl_3_ | 0.53 ± 0.23 | 4.8 ± 3.83 | 39.31 ± 13.04 | > 50 | > 50**** |
| [^161^Tb]Tb-**DTPA**-HSA | 0.07 ± 0.03 | 0.1 ± 0.02 | 0.12 ± 0.04 | 0.42 ± 0.05 | > 50 |
| [^161^Tb]Tb-**DOTA**-HSA | 0.27 ± 0.36 | 0.1 ± 0.09 | 0.05 ± 0.03 | 0.24 ± 0.09 | 5.61 ± 2.00 |
| [^161^Tb]Tb-**DOTA-GA**-HSA | 0.05 ± 0.00 | 0.05 ± 0.01 | 0.06 ± 0.00 | 0.14 ± 0.01 | 1.66 ± 0.50 |
| [^161^Tb]Tb-**NETA**-HSA | 0.08 ± 0.00 | 0.06 ± 0.00 | 0.11 ± 0.02 | 0.29 ± 0.07 | 11.23 ± 2.18 |

| Bone-to-muscle | 10 min | 1 hr | 4 hr | 24 hr | 7 d |
| --- | --- | --- | --- | --- | --- |
| ^161^TbCl_3_ | 3.43 ± 0.94 | 17.01 ± 8.76 | 39.68 ± 11.71**** | 41.95 ± 7.91*** | > 50 **** |
| [^161^Tb]Tb-**DTPA**-HSA | 3.77 ± 0.93 | 4.27 ± 0.65 | 2.26 ± 0.69 | 2.21 ± 0.72 | 6.36 ± 0.63 |
| [^161^Tb]Tb-**DOTA**-HSA | 3.41 ± 2.02 | 1.82 ± 0.94 | 1.9 ± 1.37 | 1.81 ± 1.08 | 3.92 ± 0.27 |
| [^161^Tb]Tb-**DOTA-GA**-HSA | 3.00 ± 0.46 | 2.46 ± 0.13 | 1.52 ± 0.12 | 1.13 ± 0.43 | 1.87 ± 0.85 |
| [^161^Tb]Tb-**NETA**-HSA | 3.87 ± 0.2 | 3.31 ± 1.15 | 1.63 ± 0.28 | 1.3 ± 0.21 | 8.49 ± 3.15 |

**Table S3.** DOTA reaction with ^nat^TbCl_3_ (1:1 molar equivalent) for 60 minutes at 2 different pH. Reaction conversion was determined by UV-LC-HRMS.

| pH of 0.1M sodium acetate/acetic acid | Reaction conversion (%) |
| --- | --- |
| 4.7 | 99 |
| 2.0 | 0 |

**Table S4.** %ID of [^161^Tb]TbCl_3_ in healthy mice

|  | **10 min** | **60 min** | **4 h** | **24 h** | **7 d** |
| --- | --- | --- | --- | --- | --- |
| blood | 24.7 ± 7.9 | 6.2 ± 4.8 | 1.0 ± 0.3 | 0.2 ± 0.2 | 0.0 ± 0.0 |
| bone | 21.2 ± 9.1 | 30.9 ± 5.5 | 60.2 ± 6.4 | 55.5 ± 14.5 | 42.4 ± 12.2 |
| liver | 9.5 ± 3.2 | 15.2 ± 3.9 | 23.8 ± 2.5 | 7.2 ± 2.0 | 5.5 ± 2.7 |
| kidneys | 3.8 ± 0.4 | 3.1 ± 1.6 | 3.5 ± 1.2 | 1.8 ± 0.3 | 0.6 ± 0.2 |
| lungs | 1.7 ± 0.6 | 1.0 ± 0.5 | 0.4 ± 0.0 | 0.2 ± 0.1 | 0.1 ± 0.0 |
| heart | 0.6 ± 0.1 | 0.2 ± 0.1 | 0.3 ± 0.0 | 0.1 ± 0.0 | 0.1 ± 0.0 |
| spleen | 0.6 ± 0.3 | 0.2 ± 0.1 | 0.2 ± 0.0 | 0.1 ± 0.0 | 0.1 ± 0.0 |
| pancreas | 0.3 ± 0.1 | 0.2 ± 0.0 | 0.2 ± 0.2 | 0.1 ± 0.0 | 0.0 ± 0.0 |
| brain | 0.1 ± 0.0 | 0.0 ± 0.0 | 0.0 ± 0.0 | 0.0 ± 0.0 | 0.0 ± 0.0 |
| intestines | 3.9 ± 0.8 | 2.8 ± 1.0 | 4.3 ± 1.6 | 2.2 ± 0.4 | 0.5 ± 0.0 |
| stomach | 0.6 ± 0.1 | 0.6 ± 0.3 | 0.9 ± 0.4 | 0.4 ± 0.0 | 0.1 ± 0.0 |
| muscle | 10.5 ± 9.1 | 7.4 ± 4.0 | 5.5 ± 2.2 | 2.6 ± 2.3 | 1.5 ± 0.1 |

**Table S5.** %ID/g of [^161^Tb]TbCl_3_ in healthy mice

|  | **10 min** | **60 min** | **4 h** | **24 h** | **7 d** |
| --- | --- | --- | --- | --- | --- |
| kidneys | 8.0 ± 0.7 | 7.2 ± 4.2 | 6.8 ± 2.8 | 2.9 ± 0.5 | 0.7 ± 0.1 |
| liver | 5.6 ± 1.7 | 9.7 ± 2.9 | 11.1 ± 1.2 | 3.4 ± 0.9 | 2.6 ± 0.9 |
| spleen | 5.9 ± 3.2 | 2.5 ± 1.6 | 2.0 ± 1.3 | 1.2 ± 0.5 | 0.5 ± 0.2 |
| pancreas | 2.1 ± 1.1 | 1.0 ± 0.4 | 1.0 ± 0.9 | 0.3 ± 0.1 | 0.1 ± 0.0 |
| lungs | 7.1 ± 2.6 | 4.6 ± 3.1 | 2.0 ± 0.4 | 0.8 ± 0.5 | 0.4 ± 0.1 |
| heart | 4.3 ± 0.6 | 1.9 ± 1 | 1.8 ± 0.1 | 0.8 ± 0.1 | 0.3 ± 0.0 |
| brain | 0.3 ± 0.1 | 0.1 ± 0.1 | 0.1 ± 0.0 | 0.1 ± 0.0 | 0.0 ± 0.0 |
| blood | 0.0 ± 0.0 | 0.0 ± 0.0 | 0.0 ± 0.0 | 0.0 ± 0.0 | 0.0 ± 0.0 |
| bone | 0.0 ± 0.0 | 0.0 ± 0.0 | 0.0 ± 0.0 | 0.0 ± 0.0 | 0.0 ± 0.0 |
| muscle | 9.7 ± 3.5 | 2.3 ± 1.9 | 0.4 ± 0.1 | 0.1 ± 0.1 | 0.0 ± 0.0 |

**Table S6.** SUV of [^161^Tb]TbCl_3_ in healthy mice

|  | **10 min** | **60 min** | **4 h** | **24 h** | **7 d** |
| --- | --- | --- | --- | --- | --- |
| blood | 3.5 ± 1.1 | 0.9 ± 0.7 | 0.1 ± 0.0 | 0.0 ± 0.0 | 0.0 ± 0.0 |
| bone | 1.8 ± 0.8 | 2.6 ± 0.5 | 5.0 ± 0.5 | 4.6 ± 1.2 | 3.5 ± 1.0 |
| lungs | 2.6 ± 0.8 | 1.7 ± 1.2 | 0.8 ± 0.2 | 0.3 ± 0.2 | 0.2 ± 0.0 |
| heart | 1.6 ± 0.1 | 0.7 ± 0.4 | 0.7 ± 0.1 | 0.3 ± 0.0 | 0.2 ± 0.1 |
| kidneys | 3.0 ± 0.2 | 2.7 ± 1.5 | 2.5 ± 1.1 | 1.1 ± 0.2 | 0.4 ± 0.2 |
| liver | 2.1 ± 0.7 | 3.7 ± 1.3 | 4.1 ± 0.4 | 1.3 ± 0.4 | 1.4 ± 0.8 |
| spleen | 2.2 ± 1.2 | 1.0 ± 0.6 | 0.7 ± 0.5 | 0.4 ± 0.2 | 0.3 ± 0.2 |
| pancreas | 0.8 ± 0.4 | 0.4 ± 0.1 | 0.4 ± 0.3 | 0.1 ± 0.1 | 0.1 ± 0.0 |
| brain | 0.1 ± 0.0 | 0.0 ± 0.0 | 0.0 ± 0.0 | 0.0 ± 0.0 | 0.0 ± 0.0 |
| muscle | 0.3 ± 0.2 | 0.2 ± 0.1 | 0.1 ± 0.1 | 0.1 ± 0.1 | 0.0 ± 0.0 |

**Table S7.** %ID of [^161^Tb]Tb-DTPA-HSA in healthy mice

|  | **10 min** | **60 min** | **4 h** | **24 h** | **7 d** |
| --- | --- | --- | --- | --- | --- |
| blood | 39.4 ± 10.6 | 45.1 ± 1.7 | 35.7 ± 2.5 | 13.6 ± 3 | 0.1 ± 0.0 |
| bone | 5.3 ± 3.6 | 7.7 ± 1.7 | 7.4 ± 2.6 | 10 ± 3.1 | 13.2 ± 4.1 |
| liver | 6.9 ± 2.4 | 8.4 ± 1.6 | 9.8 ± 0.3 | 11.8 ± 2.8 | 5.6 ± 0.3 |
| kidneys | 2.3 ± 0.8 | 2.9 ± 0.5 | 3.0 ± 0.1 | 3.2 ± 0.9 | 0.8 ± 0.2 |
| lungs | 2.2 ± 1.9 | 3.1 ± 1.1 | 1.9 ± 0.2 | 1.5 ± 1.0 | 0.3 ± 0.1 |
| heart | 0.6 ± 0.4 | 0.8 ± 0.1 | 0.8 ± 0.2 | 0.7 ± 0.2 | 0.2 ± 0.0 |
| spleen | 0.3 ± 0.1 | 0.3 ± 0.1 | 0.4 ± 0.1 | 0.5 ± 0.1 | 0.3 ± 0.1 |
| pancreas | 0.1 ± 0.0 | 0.2 ± 0.0 | 0.3 ± 0.0 | 0.5 ± 0.2 | 0.1 ± 0.0 |
| brain | 0.3 ± 0.1 | 0.3 ± 0.1 | 0.2 ± 0.0 | 0.1 ± 0.0 | 0.0 ± 0.0 |
| intestines | 2.8 ± 1.1 | 5.7 ± 2.3 | 9.0 ± 0.5 | 5.2 ± 1.3 | 1.4 ± 0.2 |
| stomach | 0.2 ± 0.0 | 0.3 ± 0.1 | 0.6 ± 0.2 | 0.7 ± 0.3 | 0.2 ± 0.0 |
| muscle | 4.5 ± 2.2 | 6.0 ± 0.9 | 10.9 ± 1.0 | 15.1 ± 2.0 | 7.0 ± 2.6 |

**Table S8.** %ID/g of [^161^Tb]Tb-DTPA-HSA in healthy mice

|  | **10 min** | **60 min** | **4 h** | **24 h** | **7 d** |
| --- | --- | --- | --- | --- | --- |
| kidneys | 5.0 ± 2.2 | 6.5 ± 1.8 | 5.5 ± 0.6 | 5.2 ± 1.4 | 1.4 ± 0.3 |
| liver | 4.1 ± 1.5 | 5.4 ± 1.2 | 4.6 ± 0.2 | 5.6 ± 1.2 | 2.9 ± 0.3 |
| spleen | 2.8 ± 1.4 | 4.0 ± 0.9 | 5.0 ± 1.1 | 5.4 ± 1.8 | 3.7 ± 0.7 |
| pancreas | 0.6 ± 0.4 | 0.9 ± 0.1 | 1.4 ± 0.1 | 2.2 ± 0.6 | 0.7 ± 0.1 |
| lungs | 8.5 ± 5.9 | 12.3 ± 1.7 | 9.1 ± 1.0 | 4.5 ± 1.4 | 1.2 ± 0.5 |
| heart | 4.5 ± 2.4 | 6.5 ± 0.8 | 5.8 ± 1.3 | 4.3 ± 1.2 | 1.4 ± 0.2 |
| brain | 0.7 ± 0.4 | 0.7 ± 0.3 | 0.5 ± 0.1 | 0.2 ± 0.1 | 0.0 ± 0.0 |
| blood | 19.5 ± 5.9 | 22.7 ± 0.5 | 13.8 ± 0.6 | 5.6 ± 1.5 | 0.0 ± 0.0 |
| bone | 1.6 ± 1.1 | 2.3 ± 0.5 | 1.7 ± 0.5 | 2.4 ± 0.9 | 3.0 ± 0.6 |
| muscle | 0.4 ± 0.2 | 0.5 ± 0.1 | 0.7 ± 0.1 | 1.1 ± 0.2 | 0.5 ± 0.1 |

**Table S9.** SUV of [^161^Tb]Tb-DTPA-HSA in healthy mice

|  | **10 min** | **60 min** | **4 h** | **24 h** | **7 d** |
| --- | --- | --- | --- | --- | --- |
| blood | 5.6 ± 1.5 | 6.4 ± 0.2 | 5.1 ± 0.4 | 1.9 ± 0.4 | 0.0 ± 0.0 |
| bone | 0.4 ± 0.3 | 0.6 ± 0.1 | 0.6 ± 0.2 | 0.8 ± 0.3 | 1.1 ± 0.3 |
| lungs | 2.4 ± 1.6 | 3.5 ± 0.4 | 3.4 ± 0.4 | 1.6 ± 0.4 | 0.4 ± 0.2 |
| heart | 1.3 ± 0.7 | 1.8 ± 0.2 | 2.2 ± 0.5 | 1.5 ± 0.3 | 0.5 ± 0.1 |
| kidneys | 1.4 ± 0.6 | 1.8 ± 0.5 | 2.1 ± 0.3 | 1.8 ± 0.4 | 0.5 ± 0.2 |
| liver | 1.2 ± 0.4 | 1.5 ± 0.3 | 1.7 ± 0.1 | 1.9 ± 0.4 | 1.0 ± 0.2 |
| spleen | 0.8 ± 0.4 | 1.1 ± 0.2 | 1.9 ± 0.5 | 1.9 ± 0.6 | 1.3 ± 0.4 |
| pancreas | 0.2 ± 0.1 | 0.2 ± 0.0 | 0.5 ± 0.0 | 0.7 ± 0.2 | 0.2 ± 0.1 |
| brain | 0.2 ± 0.1 | 0.2 ± 0.1 | 0.2 ± 0.0 | 0.1 ± 0.0 | 0.0 ± 0.0 |
| muscle | 0.1 ± 0.1 | 0.1 ± 0.0 | 0.3 ± 0.0 | 0.4 ± 0.1 | 0.2 ± 0.1 |

**Table S10.** %ID of [^161^Tb]Tb-DOTA-HSA in healthy mice

|  | **10 min** | **60 min** | **4 h** | **24 h** | **7 d** |
| --- | --- | --- | --- | --- | --- |
| blood | 49.9 ± 4.3 | 49.6 ± 21.6 | 38.7 ± 9.3 | 6.7 ± 2.8 | 0.2 ± 0.1 |
| bone | 23.9 ± 31.9 | 6.1 ± 3.2 | 3.2 ± 1.8 | 2.5 ± 0.2 | 1.6 ± 0.5 |
| liver | 6.3 ± 0.5 | 7.0 ± 0.9 | 5.5 ± 0.4 | 4.0 ± 0.5 | 2.1 ± 0.1 |
| kidneys | 2.4 ± 0.1 | 2.3 ± 0.3 | 1.9 ± 0.6 | 0.9 ± 0.1 | 0.1 ± 0.0 |
| lungs | 1.5 ± 0.2 | 2.8 ± 1.7 | 1.5 ± 0.2 | 0.8 ± 0.2 | 0.1 ± 0.0 |
| heart | 0.7 ± 0.2 | 0.7 ± 0.1 | 0.7 ± 0.1 | 0.4 ± 0.0 | 0.1 ± 0.0 |
| spleen | 0.3 ± 0.0 | 0.3 ± 0.1 | 0.5 ± 0.3 | 0.2 ± 0.0 | 0.2 ± 0.0 |
| pancreas | 0.2 ± 0.1 | 0.4 ± 0.4 | 0.2 ± 0.0 | 0.3 ± 0.1 | 0.1 ± 0.0 |
| brain | 0.4 ± 0.1 | 0.2 ± 0.0 | 0.2 ± 0.0 | 0.1 ± 0.0 | 0.0 ± 0.0 |
| intestines | 2.0 ± 0.4 | 3.8 ± 0.4 | 3.6 ± 0.1 | 2.4 ± 0.1 | 0.5 ± 0.1 |
| stomach | 0.2 ± 0.0 | 0.3 ± 0.1 | 0.4 ± 0.0 | 0.2 ± 0.0 | 0.1 ± 0.0 |
| muscle | 4.6 ± 1.6 | 13.2 ± 10.1 | 7.9 ± 4.7 | 5.4 ± 2.2 | 1.3 ± 0.3 |

**Table S11.** %ID/g of [^161^Tb]Tb-DOTA-HSA in healthy mice

|  | **10 min** | **60 min** | **4 h** | **24 h** | **7 d** |
| --- | --- | --- | --- | --- | --- |
| kidneys | 4.2 ± 0.5 | 4.7 ± 0.4 | 2.7 ± 0.7 | 1.6 ± 0.1 | 0.2 ± 0.0 |
| liver | 3.1 ± 0.4 | 3.7 ± 0.6 | 2.5 ± 0.0 | 2.2 ± 0.3 | 0.8 ± 0.1 |
| spleen | 2.8 ± 0.5 | 3.0 ± 1.7 | 2.1 ± 0.5 | 2.1 ± 0.7 | 1.5 ± 0.3 |
| pancreas | 0.6 ± 0.1 | 1.5 ± 1.7 | 0.8 ± 0.0 | 0.8 ± 0.1 | 0.2 ± 0.1 |
| lungs | 5.6 ± 1.2 | 14.9 ± 11 | 5.2 ± 0.6 | 2.6 ± 0.5 | 0.2 ± 0.0 |
| heart | 5.1 ± 1.8 | 4.8 ± 1.8 | 3.1 ± 0.4 | 2.1 ± 0.2 | 0.3 ± 0.1 |
| brain | 0.8 ± 0.3 | 0.5 ± 0.1 | 0.4 ± 0.1 | 0.2 ± 0.0 | 0.0 ± 0.0 |
| blood | 21.7 ± 2.9 | 21.2 ± 9.0 | 12.1 ± 2.7 | 2.5 ± 1.1 | 0.1 ± 0.0 |
| bone | 1.4 ± 0.8 | 1.5 ± 0.8 | 0.6 ± 0.3 | 0.5 ± 0.0 | 0.3 ± 0.1 |
| muscle | 0.3 ± 0.1 | 1.0 ± 0.8 | 0.4 ± 0.2 | 0.4 ± 0.2 | 0.1 ± 0.0 |

**Table S12.** SUV of [^161^Tb]Tb-DOTA-HSA in healthy mice

|  | **10 min** | **60 min** | **4 h** | **24 h** | **7 d** |
| --- | --- | --- | --- | --- | --- |
| blood | 7.1 ± 0.6 | 7.1 ± 3.1 | 5.5 ± 1.3 | 1.0 ± 0.4 | 0.0 ± 0.0 |
| bone | 2.0 ± 2.7 | 0.5 ± 0.3 | 0.3 ± 0.1 | 0.2 ± 0.0 | 0.1 ± 0.0 |
| lungs | 1.9 ± 0.5 | 5.0 ± 3.6 | 2.4 ± 0.2 | 1.0 ± 0.2 | 0.1 ± 0.0 |
| heart | 1.7 ± 0.5 | 1.6 ± 0.6 | 1.4 ± 0.2 | 0.8 ± 0.1 | 0.1 ± 0.0 |
| kidneys | 1.4 ± 0.1 | 1.6 ± 0.1 | 1.2 ± 0.3 | 0.6 ± 0.0 | 0.1 ± 0.0 |
| liver | 1.0 ± 0.1 | 1.2 ± 0.2 | 1.1 ± 0.0 | 0.8 ± 0.1 | 0.4 ± 0.0 |
| spleen | 0.9 ± 0.1 | 1.0 ± 0.6 | 1.0 ± 0.2 | 0.8 ± 0.3 | 0.7 ± 0.1 |
| pancreas | 0.2 ± 0.0 | 0.5 ± 0.6 | 0.4 ± 0.0 | 0.3 ± 0.0 | 0.1 ± 0.0 |
| brain | 0.3 ± 0.1 | 0.2 ± 0.0 | 0.2 ± 0.0 | 0.1 ± 0.0 | 0.0 ± 0.0 |
| muscle | 0.1 ± 0.0 | 0.3 ± 0.3 | 0.2 ± 0.1 | 0.1 ± 0.1 | 0.0 ± 0.0 |

**Table S13.** %ID of [^161^Tb]Tb-DOTA-GA-HSA in healthy mice

|  | **10 min** | **60 min** | **4 h** | **24 h** | **7 d** |
| --- | --- | --- | --- | --- | --- |
| blood | 43.6 ± 4.1 | 39.6 ± 7.0 | 34.8 ± 3.1 | 13.5 ± 1.7 | 0.5 ± 0.1 |
| bone | 3.5 ± 0.6 | 3.4 ± 0.3 | 3.5 ± 0.3 | 3.3 ± 0.4 | 1.5 ± 0.2 |
| liver | 5.7 ± 0.5 | 5.5 ± 0.3 | 5.6 ± 0.7 | 5.7 ± 0.5 | 3.2 ± 0.6 |
| kidneys | 2.1 ± 0.2 | 2.4 ± 0.2 | 1.7 ± 0.5 | 1.4 ± 0.1 | 0.3 ± 0.0 |
| lungs | 2.2 ± 1.2 | 2.6 ± 0.1 | 1.9 ± 1.0 | 1.2 ± 0.5 | 0.1 ± 0.0 |
| heart | 0.6 ± 0.1 | 0.8 ± 0.1 | 0.7 ± 0.1 | 0.5 ± 0.1 | 0.1 ± 0.0 |
| spleen | 0.4 ± 0.1 | 0.3 ± 0.0 | 0.3 ± 0.1 | 0.3 ± 0.0 | 0.2 ± 0.0 |
| pancreas | 0.2 ± 0.0 | 0.2 ± 0.0 | 0.3 ± 0.1 | 0.3 ± 0.1 | 0.1 ± 0.0 |
| brain | 0.2 ± 0.1 | 0.2 ± 0.1 | 0.2 ± 0.0 | 0.1 ± 0.0 | 0.0 ± 0.0 |
| intestines | 1.8 ± 0.2 | 3.0 ± 0.2 | 4.0 ± 0.5 | 3.3 ± 0.2 | 0.9 ± 0.2 |
| stomach | 0.3 ± 0.1 | 0.3 ± 0.0 | 0.5 ± 0.1 | 0.5 ± 0.1 | 0.1 ± 0.0 |
| muscle | 3.9 ± 0.9 | 4.6 ± 0.5 | 7.6 ± 0.5 | 10.8 ± 3.8 | 3.1 ± 1.6 |

**Table S14.** %ID/g of [^161^Tb]Tb-DOTA-GA-HSA in healthy mice

|  | **10 min** | **60 min** | **4 h** | **24 h** | **7 d** |
| --- | --- | --- | --- | --- | --- |
| kidneys | 2.9 ± 0.2 | 2.9 ± 0.1 | 2.4 ± 0.6 | 1.7 ± 0.0 | 0.3 ± 0.0 |
| liver | 2.4 ± 0.4 | 2.5 ± 0.2 | 2.7 ± 0.5 | 2.6 ± 0.2 | 1.4 ± 0.1 |
| spleen | 2.0 ± 0.2 | 2.1 ± 0.1 | 2.2 ± 0.6 | 2.4 ± 0.2 | 1.7 ± 0.2 |
| pancreas | 0.6 ± 0.3 | 0.6 ± 0.1 | 0.8 ± 0.2 | 1.2 ± 0.2 | 0.3 ± 0.0 |
| lungs | 7.1 ± 3.4 | 8.1 ± 0.2 | 5.8 ± 1.9 | 3.3 ± 0.1 | 0.4 ± 0.0 |
| heart | 2.9 ± 0.6 | 3.3 ± 0.1 | 3.1 ± 0.4 | 2.5 ± 0.3 | 0.6 ± 0.1 |
| brain | 0.5 ± 0.1 | 0.4 ± 0.1 | 0.4 ± 0.1 | 0.2 ± 0.0 | 0.0 ± 0.0 |
| blood | 13.5 ± 0.9 | 12.7 ± 2.0 | 10.0 ± 1.4 | 4.4 ± 0.4 | 0.2 ± 0.0 |
| bone | 0.6 ± 0.1 | 0.6 ± 0.0 | 0.6 ± 0.1 | 0.6 ± 0.0 | 0.3 ± 0.1 |
| muscle | 0.2 ± 0.0 | 0.3 ± 0.0 | 0.4 ± 0.1 | 0.6 ± 0.2 | 0.2 ± 0.1 |

**Table S15.** SUV of [^161^Tb]Tb-DOTA-GA-HSA in healthy mice

|  | **10 min** | **60 min** | **4 h** | **24 h** | **7 d** |
| --- | --- | --- | --- | --- | --- |
| blood | 6.2 ± 0.6 | 5.7 ± 1.0 | 5.0 ± 0.4 | 1.9 ± 0.2 | 0.1 ± 0.0 |
| bone | 0.3 ± 0.0 | 0.3 ± 0.0 | 0.3 ± 0.0 | 0.3 ± 0.0 | 0.1 ± 0.0 |
| lungs | 3.3 ± 1.5 | 3.6 ± 0.3 | 2.8 ± 0.5 | 1.4 ± 0.1 | 0.2 ± 0.0 |
| heart | 1.3 ± 0.3 | 1.5 ± 0.1 | 1.6 ± 0.3 | 1.1 ± 0.2 | 0.3 ± 0.1 |
| kidneys | 1.3 ± 0.1 | 1.3 ± 0.0 | 1.1 ± 0.1 | 0.8 ± 0.1 | 0.2 ± 0.0 |
| liver | 1.1 ± 0.2 | 1.1 ± 0.1 | 1.3 ± 0.0 | 1.1 ± 0.2 | 0.7 ± 0.1 |
| spleen | 0.9 ± 0.1 | 0.9 ± 0.0 | 1.1 ± 0.1 | 1.0 ± 0.2 | 0.8 ± 0.0 |
| pancreas | 0.3 ± 0.1 | 0.3 ± 0.0 | 0.4 ± 0.0 | 0.5 ± 0.1 | 0.2 ± 0.0 |
| brain | 0.2 ± 0.1 | 0.2 ± 0.0 | 0.2 ± 0.0 | 0.1 ± 0.0 | 0.0 ± 0.0 |
| muscle | 0.1 ± 0.0 | 0.1 ± 0.0 | 0.2 ± 0.0 | 0.3 ± 0.1 | 0.1 ± 0.0 |
| intestines | 1.8 ± 0.2 | 3.0 ± 0.2 | 4.0 ± 0.5 | 3.3 ± 0.2 | 0.9 ± 0.2 |
| stomach | 0.3 ± 0.1 | 0.3 ± 0.0 | 0.5 ± 0.1 | 0.5 ± 0.1 | 0.1 ± 0.0 |
| muscle | 3.9 ± 0.9 | 4.6 ± 0.5 | 7.6 ± 0.5 | 10.8 ± 3.8 | 3.1 ± 1.6 |

**Table S16.** %ID of [^161^Tb]Tb-NETA-HSA in healthy mice

|  | **10 min** | **60 min** | **4 h** | **24 h** | **7 d** |
| --- | --- | --- | --- | --- | --- |
| blood | 44.3 ± 1.4 | 44.1 ± 6.0 | 34.5 ± 3 | 10.2 ± 1.0 | 0.1 ± 0.0 |
| bone | 4.7 ± 0.2 | 4.2 ± 0.8 | 5.8 ± 1.3 | 4.6 ± 0.7 | 2.6 ± 0.8 |
| liver | 6.1 ± 0.7 | 6.3 ± 0.9 | 6.0 ± 0.5 | 5.4 ± 0.5 | 2.3 ± 0.1 |
| kidneys | 2.1 ± 0.2 | 2.1 ± 0.5 | 1.6 ± 0.2 | 1.2 ± 0.1 | 0.1 ± 0.0 |
| lungs | 2.5 ± 1.1 | 4.2 ± 1.1 | 2.3 ± 1.5 | 0.9 ± 0.5 | 0.1 ± 0.0 |
| heart | 0.5 ± 0.1 | 1.0 ± 0.4 | 0.7 ± 0.1 | 0.4 ± 0.0 | 0.0 ± 0.0 |
| spleen | 0.2 ± 0.0 | 0.3 ± 0.1 | 0.2 ± 0.0 | 0.2 ± 0.0 | 0.1 ± 0.0 |
| pancreas | 0.1 ± 0.0 | 0.2 ± 0.0 | 0.3 ± 0.0 | 0.4 ± 0.1 | 0.0 ± 0.0 |
| brain | 0.2 ± 0.1 | 0.2 ± 0.0 | 0.1 ± 0.0 | 0.1 ± 0.0 | 0.0 ± 0.0 |
| intestines | 1.9 ± 0.5 | 3.3 ± 0.7 | 4.5 ± 0.4 | 3.8 ± 0.7 | 0.7 ± 0.1 |
| stomach | 0.2 ± 0.0 | 0.4 ± 0.2 | 0.5 ± 0.1 | 0.3 ± 0.0 | 0.1 ± 0.0 |
| muscle | 4.1 ± 0.2 | 4.6 ± 1.8 | 11.9 ± 0.9 | 12 ± 2.6 | 1.0 ± 0.2 |

**Table S17.** %ID/g of [^161^Tb]Tb-NETA-HSA in healthy mice

|  | **10 min** | **60 min** | **4 h** | **24 h** | **7 d** |
| --- | --- | --- | --- | --- | --- |
| blood | 19.1 ± 0.8 | 20.4 ± 2.9 | 15.6 ± 1.1 | 5.4 ± 0.5 | 0.1 ± 0.0 |
| bone | 1.5 ± 0.1 | 1.3 ± 0.2 | 1.8 ± 0.4 | 1.5 ± 0.3 | 1 ± 0.3 |
| brain | 0.6 ± 0.2 | 0.6 ± 0.1 | 0.5 ± 0.1 | 0.3 ± 0.0 | 0.0 ± 0.0 |
| heart | 4.0 ± 0.5 | 7.2 ± 2.9 | 5.7 ± 0.7 | 3.3 ± 0.1 | 0.5 ± 0.1 |
| kidneys | 5.0 ± 0.6 | 4.9 ± 1.0 | 3.9 ± 0.4 | 2.7 ± 0.3 | 0.5 ± 0.2 |
| liver | 3.9 ± 0.4 | 4.0 ± 0.4 | 3.9 ± 0.3 | 3.6 ± 0.3 | 2.0 ± 0.2 |
| lungs | 11.1 ± 2.2 | 13.9 ± 1.9 | 9.5 ± 1.5 | 4.1 ± 1.0 | 0.4 ± 0.1 |
| muscle | 0.4 ± 0.0 | 0.4 ± 0.2 | 1.1 ± 0.1 | 1.2 ± 0.3 | 0.1 ± 0.0 |
| pancreas | 0.7 ± 0.2 | 0.8 ± 0.2 | 1.4 ± 0.1 | 1.8 ± 0.1 | 0.3 ± 0.1 |
| spleen | 3.0 ± 0.1 | 3.3 ± 0.4 | 3.3 ± 0.3 | 2.9 ± 0.4 | 2.2 ± 0.6 |

**Table S18.** SUV of [^161^Tb]Tb-NETA-HSA in healthy mice

|  | **10 min** | **60 min** | **4 h** | **24 h** | **7 d** |
| --- | --- | --- | --- | --- | --- |
| blood | 6.3 ± 0.2 | 6.3 ± 0.9 | 4.9 ± 0.4 | 1.5 ± 0.1 | 0.0 ± 0.0 |
| bone | 0.4 ± 0.0 | 0.4 ± 0.1 | 0.5 ± 0.1 | 0.4 ± 0.1 | 0.2 ± 0.1 |
| lungs | 3.1 ± 0.7 | 4.0 ± 0.6 | 2.7 ± 0.4 | 1.0 ± 0.3 | 0.1 ± 0.0 |
| heart | 1.1 ± 0.1 | 2.0 ± 0.8 | 1.6 ± 0.2 | 0.8 ± 0.0 | 0.1 ± 0.0 |
| kidneys | 1.3 ± 0.2 | 1.3 ± 0.3 | 1.1 ± 0.1 | 0.7 ± 0.1 | 0.1 ± 0.0 |
| liver | 1.2 ± 0.1 | 1.2 ± 0.2 | 1.2 ± 0.1 | 1.0 ± 0.1 | 0.4 ± 0.0 |
| spleen | 0.8 ± 0.0 | 0.9 ± 0.1 | 0.9 ± 0.1 | 0.7 ± 0.1 | 0.5 ± 0.1 |
| pancreas | 0.2 ± 0.0 | 0.2 ± 0.1 | 0.4 ± 0.0 | 0.5 ± 0.0 | 0.1 ± 0.0 |
| brain | 0.2 ± 0.0 | 0.2 ± 0.0 | 0.1 ± 0.0 | 0.1 ± 0.0 | 0.0 ± 0.0 |
| muscle | 0.1 ± 0.0 | 0.1 ± 0.0 | 0.3 ± 0.0 | 0.3 ± 0.1 | 0.0 ± 0.0 |
| intestines | 1.9 ± 0.5 | 3.3 ± 0.7 | 4.5 ± 0.4 | 3.8 ± 0.7 | 0.7 ± 0.1 |
| stomach | 0.2 ± 0.0 | 0.4 ± 0.2 | 0.5 ± 0.1 | 0.3 ± 0.0 | 0.1 ± 0.0 |
| muscle | 4.1 ± 0.2 | 4.6 ± 1.8 | 11.9 ± 0.9 | 12 ± 2.6 | 1.0 ± 0.2 |
